# Choice of Non-Inferiority (NI) Margins Does Not Protect against Degradation of Treatment Effects on an Average – An Observational Study of Registered and Published NI Trials

**DOI:** 10.1371/journal.pone.0103616

**Published:** 2014-07-31

**Authors:** Beryl Primrose Gladstone, Werner Vach

**Affiliations:** Clinical Epidemiology Group, Institute of Medical Biometry and Medical Informatics, University Medical Center Freiburg, Freiburg, Germany; Institut National de la Santé et de la Recherche Médicale, France

## Abstract

**Objective:**

NI margins have to be chosen appropriately to control the risk of degradation of treatment effects in non-inferiority (NI) trials. We aimed to study whether the current choice of NI margins protects sufficiently against a degradation of treatment effect on an average.

**Study Design and Setting:**

NI trials reflecting current practice were assembled and for each trial, the NI margin was translated into a likelihood of degradation. The likelihood of degradation was calculated as the conditional probability of a treatment being harmful given that it is declared non-inferior in the trial, using simulation. Its distribution among the NI trials was then studied to assess the potential risk of degradation.

**Results:**

The median (lower/upper quartile) NI margin among 112 binary outcome NI trials corresponded to an odds ratio of 0.57(0.45, 0.66), while among 38 NI trials with continuous outcome, to a Cohen’s d of −0.42(−0.54, −0.31) and a hazard ratio of 0.82(0.73, 0.86) among 24 survival outcome NI trials. Overall, the median likelihood of degradation was 56% (45%, 62%).

**Conclusion:**

Only two fifths of the current NI trials had a likelihood of degradation lower than 50%, suggesting that, in majority of the NI trials, there is no sufficient protection against degradation on an average. We suggest a third hurdle for the choice of NI margins, thus contributing a sufficient degree of protection.

## Introduction

Non-inferiority (NI) trials have gained popularity over the years [1]. Though there are research areas where NI trials are not acceptable as a proof of efficacy for drug approval [2]–[4], NI trials are gaining importance in certain fields, as they are today a standard part of drug approval [5]. The aim of a NI trial is to show that a new treatment is not inferior to the standard treatment below a certain pre-specified margin called non-inferiority margin. Choice of this NI margin is a critical step in conducting NI trials. FDA [6]–[8] as well as EMEA [9], had summarised the advancement of methodological discussions on this topic and framed guidelines reinforcing the choice based on two principles: controlling the probability to preserve some of the effect of the active control assumed to be present in the NI study (typically aiming at preserving at least 50% of the effect), denoted by M1 and the smallest clinically acceptable difference of the test drug compared to the active control, taking into account an implicit advantage of the new drug, denoted by M2. The narrower of the two margins should be used [8].

However, it is possible that NI margins are set relatively wide to avoid the time and costs of performing huge trials and there have been previous reports revealing the usage of wide margins in published NI trials [10]–[12]. Hence, there have been efforts on behalf of the regulatory agencies to optimise the choice of stringent margins. In addition to the guidelines, trialists are encouraged to choose standard NI margins relevant to the research area in cooperation with the regulatory agencies, through consensus of a large group of experts [8], [13]. In addition, patient-oriented approaches to choose NI margin are found in the literature [14], [15].

D’Agostino et al [16] mentioned bio-creep as a concern related to the choice of non-inferiority margins already in 2003. Biocreep is a cyclical phenomenon where a slightly inferior treatment becomes the active control for the next generation of NI trials which over time leads to degradation of efficacy of the treatment offered to patients [16]–[23]. Moreover, the fear of bio-creep expressed by US congressmen in NI trials prompted the US General Accountability Office to study the use of NI trials by FDA (2002–2009) for its approval strategy [5]. Generally speaking, these discussions about NI trials imply that some scientists feel that there may be a degradation of treatment efficacy on an average in medical fields where NI trials are popular, even in the absence of cyclicality. A recent example is the evaluation by Röhmel & Kieser [24] of the NI margin proposed by FDA in the draft guidance for treatments for nosocomial pneumonia. They suggested a second hurdle to claim non-inferiority as an effort to curb the risk of accepting “harmful treatments”.

In this paper, we address the question of how a Principal Investigator (PI) can try to control the risk of contributing to a degradation on average by choosing an appropriate NI margin, and to which degree such a control has been achieved in existing trials. We quantify this risk by the likelihood of degradation, i.e., the probability of having declared a less efficient treatment as non-inferior in a NI trial. This likelihood allows both to judge the choice of the margin in each single study as well as the overall risk of degradation on an average: If more than half of the NI trials are associated with a likelihood of degradation above 50%, we may have to fear degradation on an average. An empirical investigation of the current distribution of the likelihood of degradation, hence, forms the main part of this paper.

## Methods

### Inclusion criteria and data extraction

Our research involved determination of likelihood of degradation among a set of NI trials reflecting current practice. They were assembled from two sources: 1) all NI trials published in four major medical journals from 2005 to 2011 assuming that they represent a high standard of study methodology, conduct and reporting- the four journals were selected from the major journals with a long history of publishing high standard clinical trials; 2) NI trials registered in the trial registers from 2000 to 2007 reflecting a source less affected by publication bias. The trials from the registers were restricted till 2007 so that there was enough time for each trial to get its results published. We included NI trials studying non-inferiority of efficacy of a new drug/treatment/therapeutic procedure/diagnostic procedure as the primary objective. NI trials aimed at determining the optimal dose of a drug with no comparison with a standard drug were excluded. Vaccine trials were excluded because they typically have many primary endpoints studying various strain−/subtype- specific antibodies and often consider protective rates close to 100%. The data from registered trials were earlier used to study the overall true treatment effect in NI trials (Gladstone and Vach [25]).

The details of identification of trials from trial registers as well as the major journals as well as the data extraction are summarized in Table 1. Relevant data were extracted from the trials fulfilling the inclusion and exclusion criteria. Analyses were performed only on trials with data on the non-inferiority margin and no attempts were made to impute missing data or to investigate sensitivity of results to this incompleteness. In case of multiple primary NI comparisons done within one trial, the sample size and the control arm’s outcome measures were chosen from the comparisons with the largest sample size. Based on the scale of the outcome variable, we classified the trials into trials with continuous, binary and survival outcomes. The margin data will be presented separately for trials with binary, continuous and survival outcome variables. The margins are presented as median (upper/lower quartile) Cohen’s d (for continuous outcome variable), Risk difference (binary) or Hazard ratio (survival) unless otherwise specified.

**Table 1:** Aspects of identification of NI trials with data on margins and data extraction.

Aspects of study data		Clinical trial registers*	Major Journals
identification and extraction			
Sources:		1. National Library of Medicine (NLM)	1. New England Journal of Medicine
		clinical trials register (www.ClinicalTrials.gov)	2. British Medical Journal
		2. ISRCTN register maintained by the	3. The Lancet
		Current Controlled Trials Ltd	4. The Journal of American Medical Association
			using Web of Science
Time period:		Trials started and completed between	Trials published during
		January 2000 and December 2007	January 2005-December 2011
Search strategy:		*“non-inferiority OR noninferiority OR*	*“non-inferiority OR noninferiority OR*
		*non inferiority OR not inferior”*	*non inferiority OR inferiority OR not inferior”*
Source of margins:		Published articles, Clinical study reports	Published articles
Data extracted:		Basic details, Outcome measure, NI margin, Actual sample size used in the final analysis	
Additional	Binary	Assumed success rate in the control arm†	
information:	Continuous	Observed population standard deviation§	
	Survival	Observed number of events in the control arm	
Margins expressed as:	Binary	Risk difference‡ (Expected success rate in the experimental group -	
		Assumed success rate in the control group) or Odds Ratio||	
	Continuous	Cohen’s d‡ = Margin/Population standard deviation	
	Survival	Hazard Ratio||	

*details described in Gladstone and Vach (17).

† observed success rate used when assumed was not reported.

§ derived from available measures of variance using standard formulas (34), when not reported.

‡ expressed as values below 0.

|| expressed as values below 1.

### Likelihood of degradation

In this paper, we refer to the true difference in the efficacy measure between the new treatment and the standard/control treatment in a NI trial as the true effect. We defined the likelihood of degradation for a single trial as the probability that the new treatment has a true effect below zero given the trial declares the treatment as non-inferior. The likelihood of degradation, *l*, depends on the distribution of the true effect. This distribution can be regarded as the distribution of true effects in NI trials similar to the current trial, e.g., in the same field of medicine targeting the same or similar disease conditions. We assume this distribution to be normal with mean *μ* and standard deviation σ. We discuss the choice of these two parameters in the next section. Given these two values, the likelihood of degradation can be determined by a simulation in the following way for a given NI trial:

10,000 NI trials were simulated under the same circumstances as in the trial (data extracted from the original trial results) but with the true effect drawn from a normal distribution with mean *μ* and standard deviation σ. The sample size was always identical to the one used in the primary analysis. In case of binary outcome, we assumed the same success rate in the control arm as observed in the trial and computed a CI for the risk difference. For a continuous outcome trial, we assumed a normal distribution with a standard deviation of one and the confidence interval for the difference in mean values was computed. In case of a survival outcome trial, trials were generated by assuming an exponential distribution and a fixed follow-up period of 3 years. These assumptions have little impact on the likelihood of degradation as it, like the power of a study, depends only on the number of events. The hazard rate in the control group was chosen such that the expected number of events was equal to the observed one in the control group. Effect estimates and confidence intervals were based on a Cox model. A trial was considered successful when the lower bound of the two-sided 95% confidence interval was above the NI margin. Among the successful trials, the proportion of trials with true effect below zero was determined and defined the likelihood of degradation.

Sensitivity analyses were carried out with two additional scenarios to check the robustness of the resulting likelihood of degradation and its dependence on the underlying distribution.

### Choice of distribution of true effects

To be able to cover a wide spectrum of true effect distribution which may differ across various medical fields, three different scenarios of distribution of the true effect were selected – optimistic, moderate and pessimistic, characterised by a proportion of 50%, 69% and 84% of harmful treatments respectively. The optimistic scenario reflects a situation of no risk of degradation on an average because even if all NI trials are successful, we can expect that overall positive and negative effects cancel out. Scenarios better than the optimistic one, with lesser than 50% harmful treatments, would never pose a risk and thus not necessary to be studied in our context. The moderate scenario reflects a situation with an implicit risk of degradation if the NI trials fail to sort out enough treatments with a negative effect. The pessimistic scenario reflects a situation with a high risk of degradation on an average: Since only 16% of all NI trials started with treatments with a positive effect, only 32% or less of the NI trials are allowed to be successful if control of degradation on an average is desired. The concrete choices of *μ* and σ are shown in Table 2. The choice is based on the following considerations: In the optimistic scenario, 50% of the new treatments should be harmful, hence we have *μ* = 0. In the continuous case, σ is chosen as 0.1 such that only 2.5% of all NI trials started with a true effect above 0.2 which is a treatment effect, above which superiority trials typically become feasible with sample sizes of less than 530 in each arm to attain 90% power. In the binary case, we regard an OR of 3/2 as a magnitude, where superiority testing becomes feasible. For example, we can demonstrate with a power of 90% a difference in response rates of 0.6 vs 0.5 with 538 patients in each arm and a difference of 0.9 vs 0.857 with 1258 patients in each arm. In the case of survival, we regard a HR of 4/3 as a corresponding magnitude, as we need 508 events overall. Consequently, the standard deviation σ of the true log OR was chosen as 1/2 log 3/2 = 0.203 and of the true log HR as 1/2 log 4/3 = 0.144 for the binary and survival outcome trials respectively.

**Table 2:** Scenarios of true effect distribution used in the calculation of likelihood of degradation.

True effect distribution	Optimistic scenario	Moderate scenario	Pessimistic scenario
% true treatment effect being positive	50%	31%	16%
Continuous outcome (Cohen‘s d)			
Average true treatment effect (*μ*)	0	−0.05	−0.1
Standard deviation (σ)	0.1	0.1	0.1
Binary outcome (log odds ratio)			
Average true treatment effect (*μ*)	0	−0.1015	−0.203
Standard deviation (σ)	−0.203	−0.203	−0.203
Average true treatment effect as OR	1	0.9	0.82
Survival outcome (log hazards ratio)			
Average true treatment effect (*μ*)	0	−0.072	−0.144
Standard deviation (σ)	−0.144	−0.144	−0.144
Average true treatment effect as HR	1	0.93	0.87

### Subgroup analyses

To address some specific hypotheses about potential determinants for the choice of the margins, a series of pre-specified subgroup analyses was performed: Do some medical fields use more liberal margins than other? Does the severity of the disease condition (life threatening yes/no) or of the outcome (mortality yes/no) influence the choice? Is there a difference between drug and non-drug interventions? Do pharmaceutically sponsored trials use more stringent margins to adhere to regulatory requirements? Is there a time trend to more liberal or to more stringent margins? Moreover, among the published trials, we compared those without registration, with registration and mentioning the NI design, and with registration but not mentioning the NI design, in order to explore further potential selection mechanisms in studying the choice of margins. All analyses were done using STATA 12 [26]. The Wilcoxon test, the Cuzick’s non-parametric trend test for continuous variables and the (stratified) Kruskal Wallis test were used to derive p-values and are reported along with the median likelihood for subgroups.

## Results

### Identification of trials and NI margins

**Figure 1:**
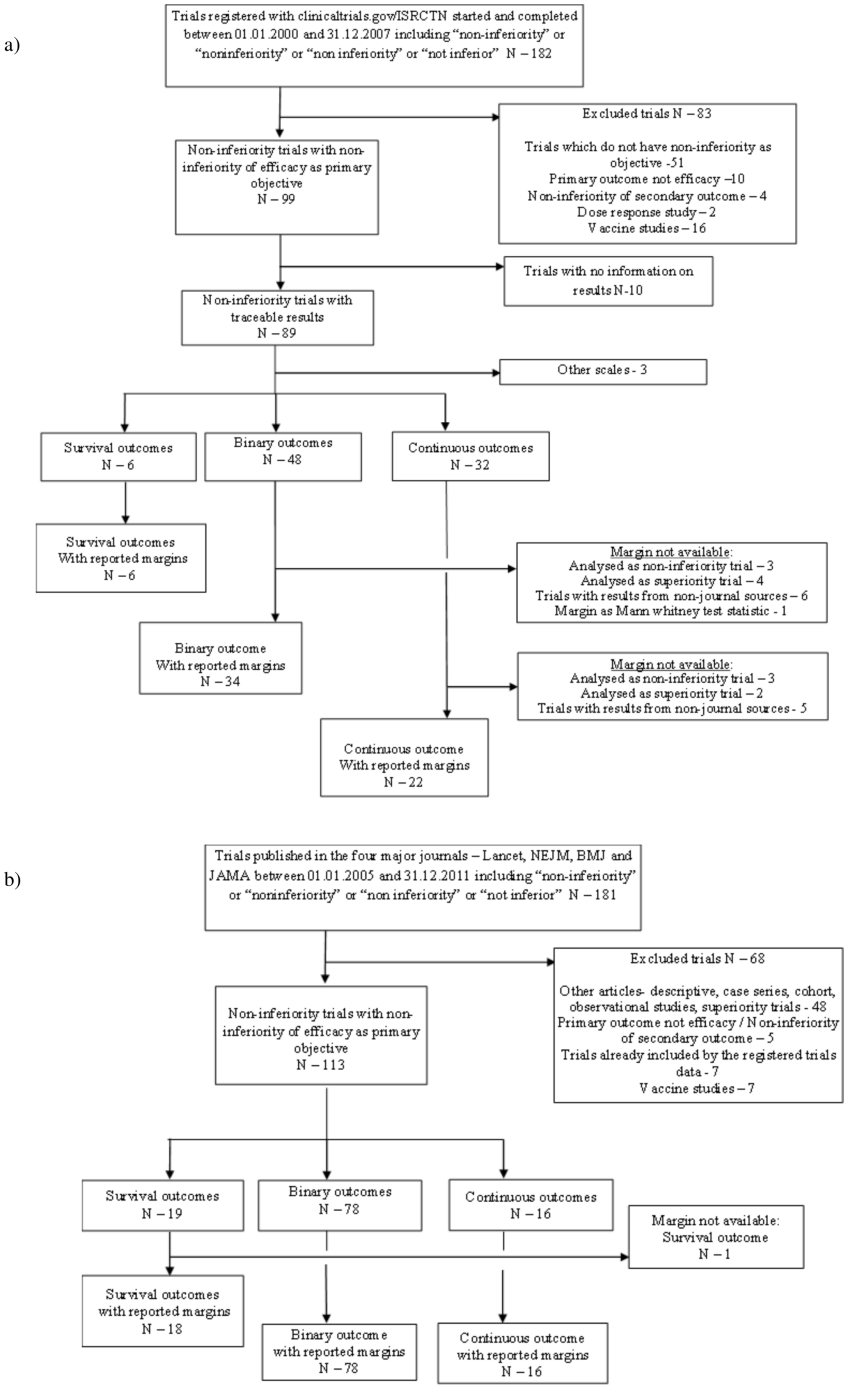
Flowchart of identification of non-inferiority trials and their margins – a) registered trials from trials register data b) published trials from four major journals.

The flow of search, exclusion and inclusion of NI trials and the NI margins among the registered and published NI trials is given in Figure 1. The computerised search in the trial registers identified 182 trials, 147 from the NLM *ClinicalTrials.gov* website and 35 from the ISRCTN website, of which 99 fulfilled the inclusion/exclusion criteria. Our search in the four major journals returned 181 results which on screening, identified original reports from 133 non-inferiority trials. 113 NI trials fulfilled the inclusion/exclusion criteria. All except one mentioned NI margin in the publication. Additional information is given in Table S1 and S2. The trial characteristics of the 174 NI trials with margins available are summarised in Table 3.

**Table 3:** Trial characteristics of the Non-inferiority trials studied.

Trial characteristics	NI trials contributing	
	NI margins (n-174)	
	n	%
**Area of research**		
Infectious diseases	45	26%
Cardiology	30	17%
Carcinoma	17	10%
Circulatory disorders	16	9%
Gastroenterology	9	5%
Psychiatry	8	5%
Diabetes	8	5%
Obstetrics & Gynecology	8	5%
Musculoskeletal	7	4%
Neurology	6	3%
Anemia, Dyslipidemia, etc.	4	2%
Hypertension	4	2%
Others	12	7%
**Source of funding**		
Industry sponsored	121	70%
Not industry sponsored	52	30%
**Type of intervention**		
Therapeutic drug interventions	134	77%
Non drug interventions	40	23%
**Source of study results**		
Registered in the two trial registers	62	36%
Published in four major journals	112	63%
**Sample size**		
Median (inter quartile range)	635 (300–1305)	
**Scale of the outcome variable**		
Binary	112	64%
Continuous	38	22%
Hazard ratio	24	14%
**Trial result as interpreted by the authors**		
Null hypothesis of inferiority rejected	134	77%
Null hypothesis of inferiority not rejected	23	13%
Superiority inferred	16	9%
Unclear	1	1%
**Year of start of the trial:**		
1993	1	1%
1996	1	1%
1997	2	1%
1998	6	3%
1999	5	3%
2000	12	7%
2001	13	7%
2002	10	6%
2003	29	17%
2004	25	14%
2005	26	15%
2006	23	13%
2007	11	6%
2008	10	6%
**Year of publication:**		
2004	1	1%
2005	17	10%
2006	18	10%
2007	29	17%
2008	25	14%
2009	30	17%
2010	35	20%
2011	19	11%

### Non-inferiority margins

**Figure 2:**
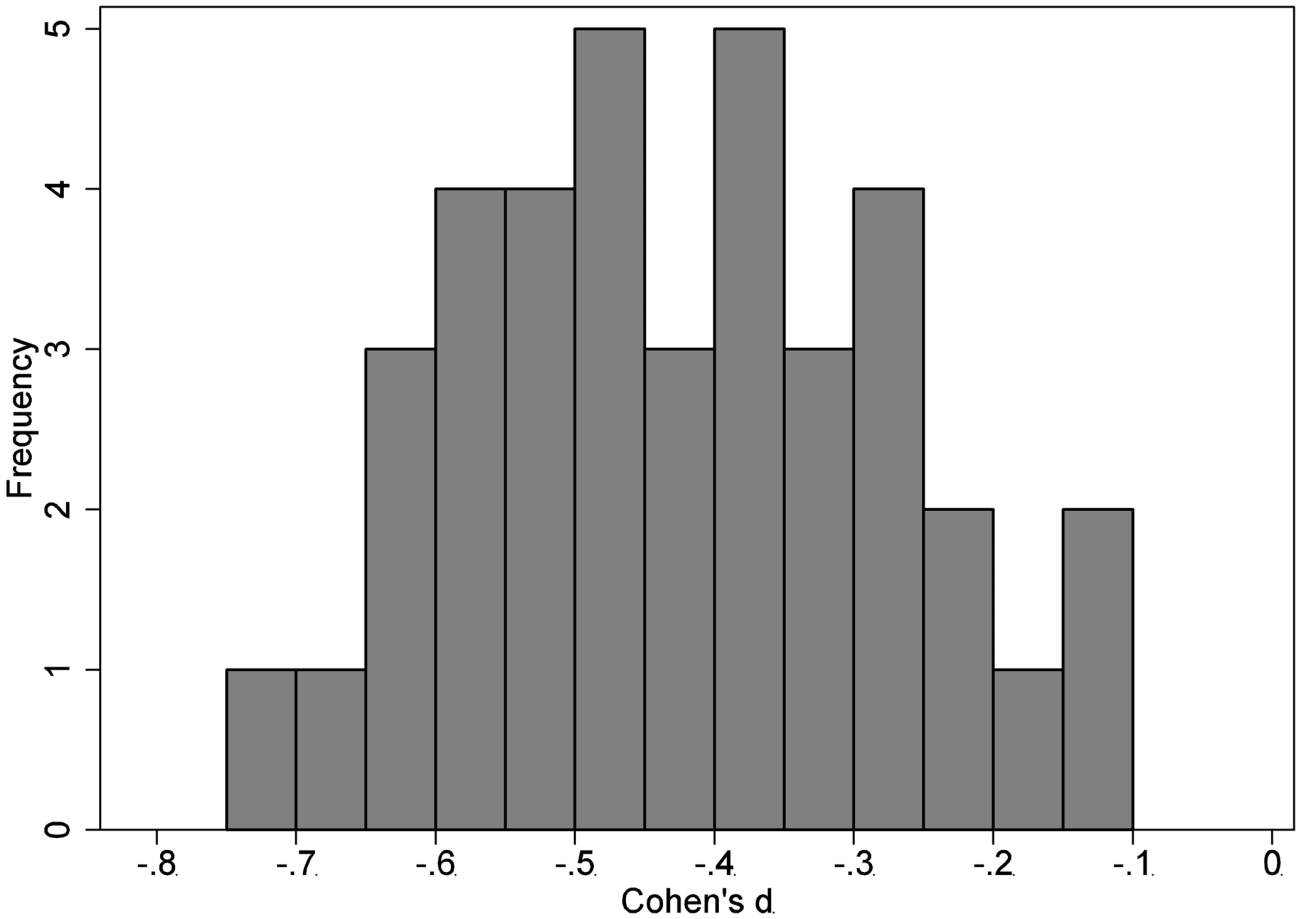
Distribution of non-inferiority margins in the NI trials for continuous outcomes.

**Figure 3:**
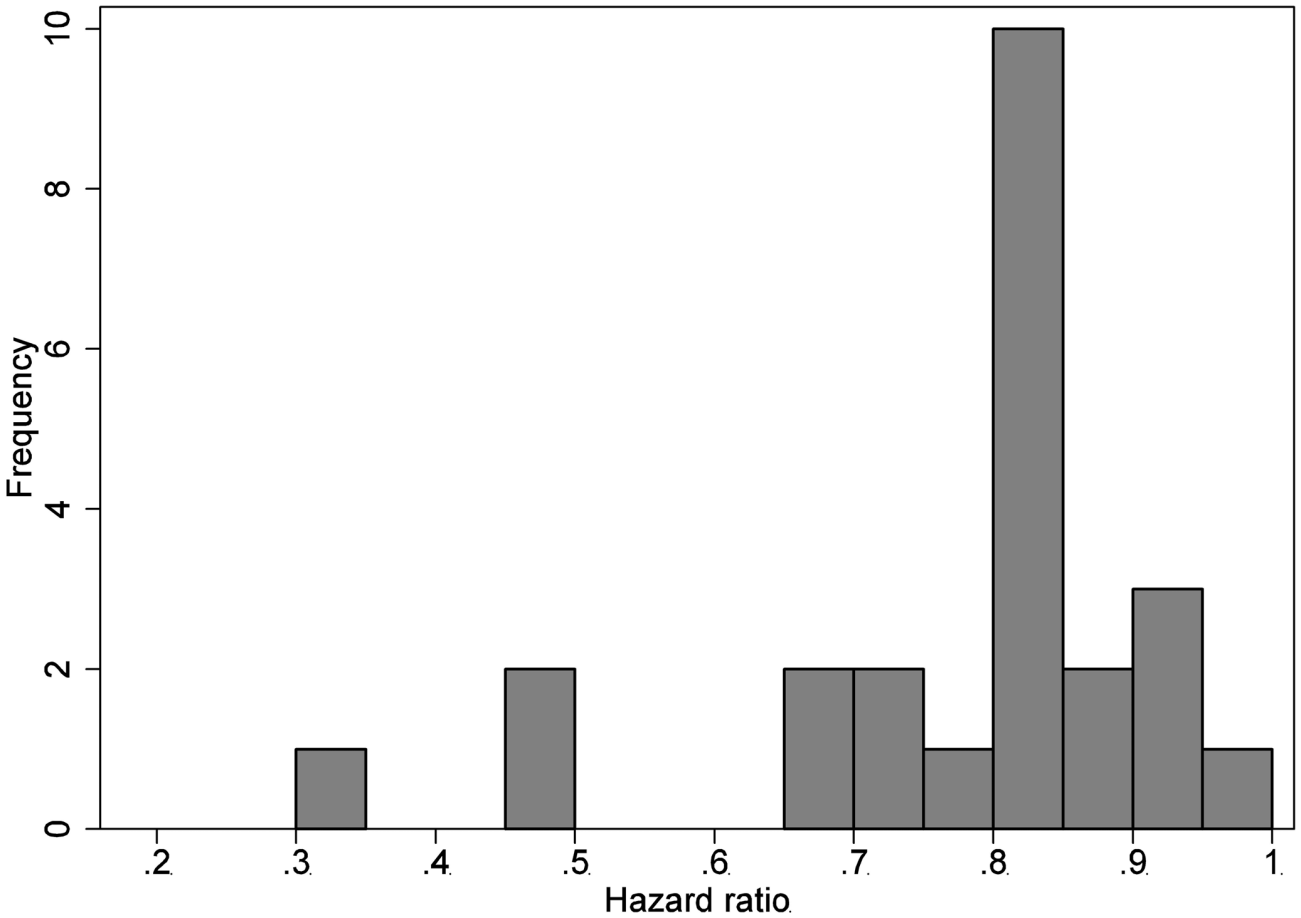
Distribution of non-inferiority margins in the NI trials for survival outcomes.

Figure 2 and 3 presents the distribution of the NI margins used in the continuous outcome and survival outcome trials respectively. The continuous outcome trials showed a median margin of Cohen’s d of −0.42(−0.54, −0.31). Among the survival outcome trials, the NI margins were always expressed as a hazard ratio and the median hazard ratio was 0.82(0.73, 0.86).

**Figure 4:**
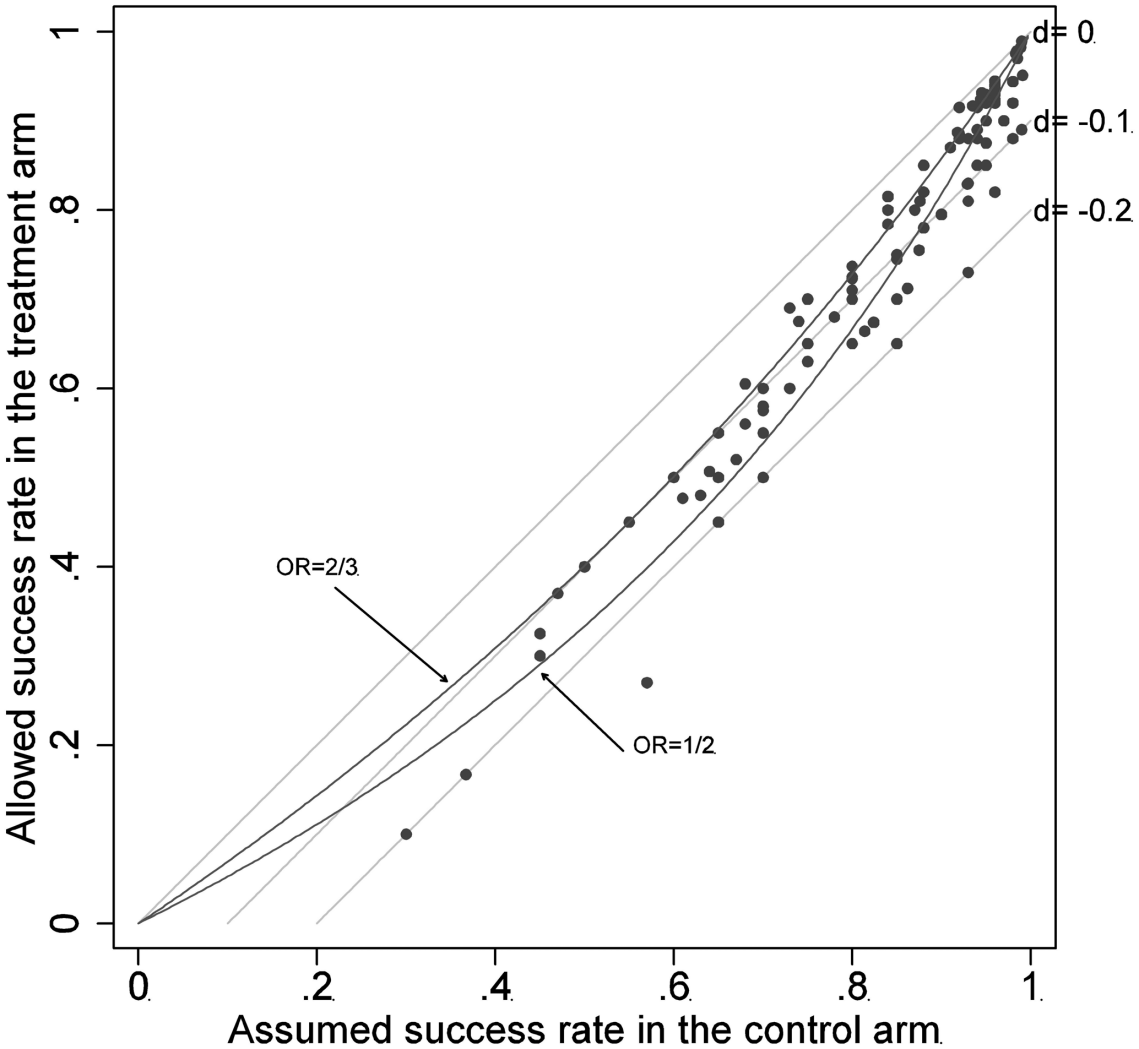
Scatterplot of the assumed success rate in the control arm versus allowed success rate in the treatment arm. The diagonal is shown as a line corresponding to a risk difference of 0. The two parallel lines correspond to risk differences of −0.1 and −0.2. The two curved lines correspond to an OR of 2/3 and 1/2.

The distribution of the margins in the binary outcome trials are shown in Figure 4 as a scatterplot of the assumed success rate in the control arm versus allowed success rate in the treatment arm. The mean (sd) success rate in the control arm was 0.82(0.15), and the median margin when expressed as a risk difference was −0.1(−0.04, −0.12) and when expressed as an OR, the median was 0.57 (0.45, 0.66). As seen in the figure, all the margins among trials with success rates in the control arm>0.8 compared to a risk difference of −0.2 or above. However, the high control success rates imply that in majority of these trials, the margins corresponded to an OR of 0.5 or below.

### Likelihood of degradation

**Figure 5:**
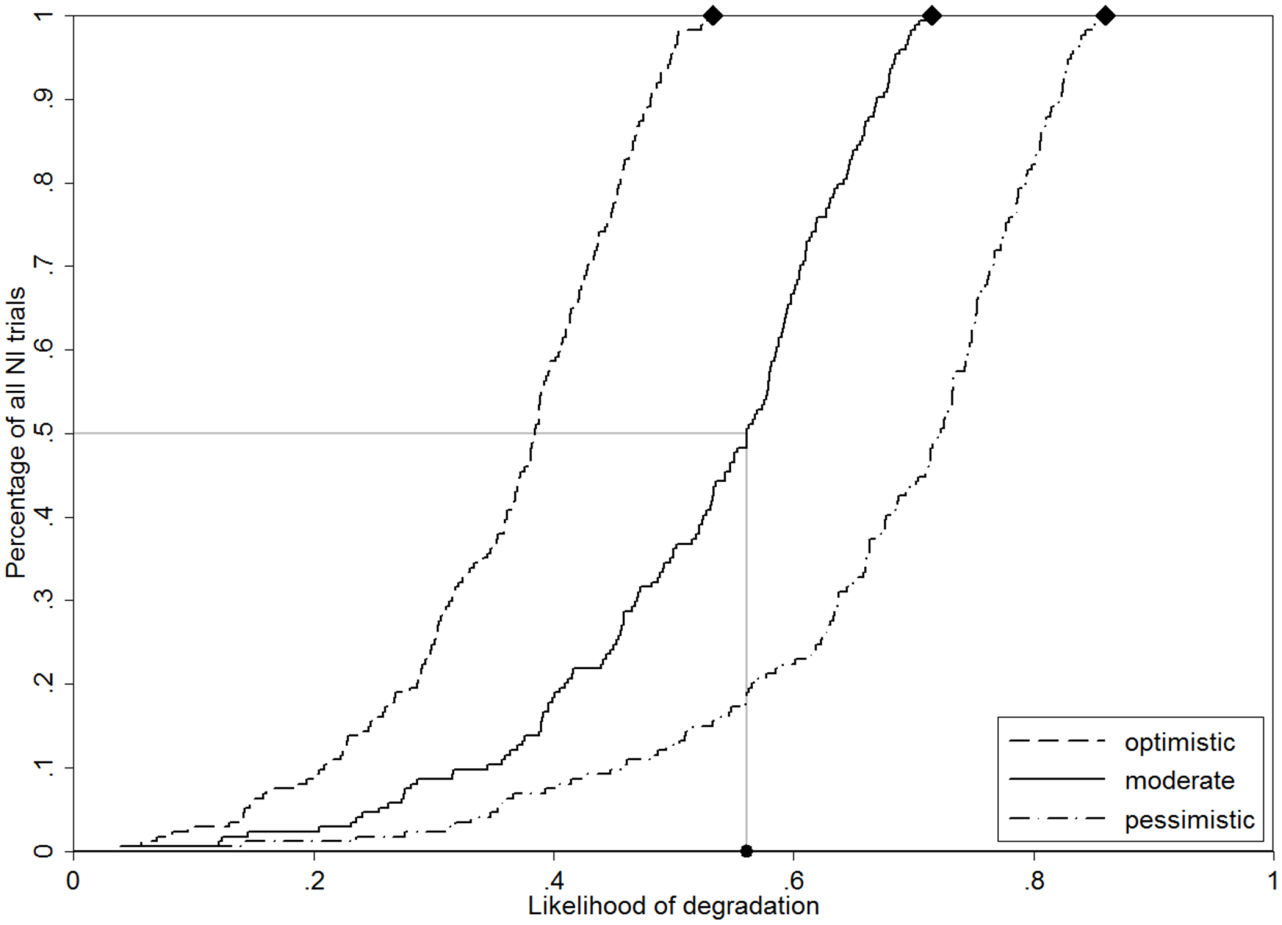
Distribution of the likelihood of degradation among the current NI trials. The diamonds represent the worst possible likelihood of degradation values and the dot represents the median likelihood of degradation in the moderate scenario.

Figure 5 presents the distribution of the likelihood of degradation among the NI trials. For each scenario, we can observe a wide range of the likelihood of degradation. While some trials have used very stringent margins translating into a likelihood of degradation less than 0.1 even under the pessimistic scenario, some have used very liberal margins such that the likelihood of degradation approaches the worst possible values. This worst possible value, namely, the probability of a harmful treatment in all NI trials independent of the result of the trial, is 0.5, 0.69 and 0.84 in the three respective scenarios (marked by diamonds in figure 4). In those trials where the likelihood of degradation approached these values, the margins chosen provided almost no protection.

It is also of interest to study the average likelihood of degradation under each scenario, as they correspond to the expected fraction of trials accepting a harmful treatment among all successful NI trials. When we compare this to the overall fraction of NI trials which started with a harmful treatment, we observe a reduction of 0.5 to 0.36 for the optimistic scenario, 0.69 to 0.52 for the moderate and 0.84 to 0.68 for the pessimistic scenario. These numbers reflect how the current practice of choosing the margin contributes to sorting out harmful treatments and hence protect against degradation on an average.

We focus only on the moderate scenario in the rest of the paper, as this reflects, in our opinion, the scenario of greatest interest. If there is some -but not an extreme- tendency to choose NI designs for harmful treatments, can we still expect to obtain a protection against degradation on an average, i.e., a likelihood of degradation less than 0.5? The median likelihood under this scenario was 0.56 and 64% have a likelihood above 0.5 suggesting that the current practice provides a protection only in about two fifth of all NI trials.

### Sensitivity analyses

We carried out sensitivity analyses studying two additional sets of scenarios by increasing *μ* and σ by 1.5 times and by decreasing them by 2/3^rd^ (Table S3). Since we kept the ratio σ/*μ* fixed, the fraction of studies with a true positive effect remained unchanged within each scenario, but the fraction of studies with very large negative or very large positive treatment effects increased or decreased respectively. There was a slight decrease or increase in the median likelihood of degradation respectively. This can be explained by the lower/higher fraction of studies with crucial decisions, i.e. with true treatment effects between the margin and 0. However, empirical distributions of the likelihood of degradation did not change substantially with the median likelihood decreasing to 0.49 and increasing to 0.62 respectively (Figure S1). The sensitivity analyses, hence, demonstrate that even with a change in the underlying distribution, the median likelihood of degradation is still close to or above 0.5.

### Trial characteristics and likelihood of degradation

**Figure 6:**
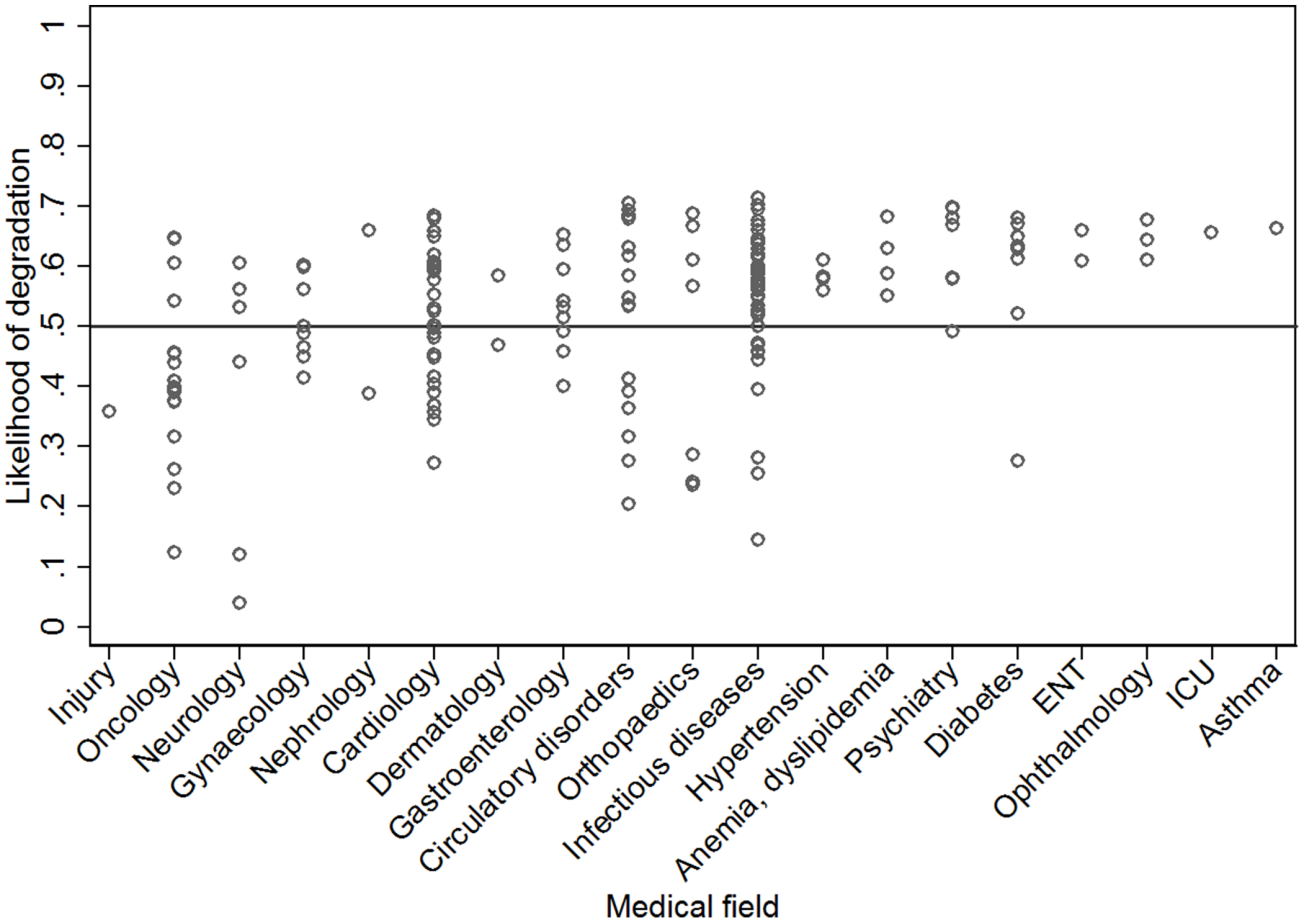
Likelihood of degradation (moderate scenario) among the NI trials stratified by medical field and sorted by the median value. o represents each trial.

NI trials on drugs for acute, life threatening diseases (n-113) had a lower median likelihood of degradation (0.53 vs 0.59, p = 0.02) compared to those related to non-acute, non-life-threatening diseases (n-61). Similarly, NI trials with mortality as the primary outcome (n-27) had a lower likelihood of degradation (0.41 vs 0.58, p<0.001) compared to those with non-mortality outcomes (n-147). There was a significant variation in likelihood of degradation (p = 0.007) among the medical fields (Figure 6). This variation could not be explained by the differences in acuteness of diseases dealt with or the primary outcome being mortality, as the variation after adjustment for these covariates, was still significant (p = 0.02). We could observe some medical fields where most of the trials exhibit a likelihood of degradation above 0.5 indicating that liberal margins are being consistently used in these fields. Considering only fields comprising of at least 4 trials each, psychiatry and diabetes had the highest median likelihood of degradation of 0.63 each while oncology had the lowest of 0.40. There was no significant trend in the likelihood of degradation based on the year of start of the study or year of publication. Neither involvement of pharmaceutical companies in sponsorship of the trial (non-pharmaceutical (n-52) = 0.56 vs pharmaceutical sponsorships (n-121) = 0.56) nor type of intervention (drug (n-134) = 0.57 vs non-drug interventions (n-40) = 0.53) was significantly associated with likelihood of degradation. Of the 112 trials from the major journals, 21 trials mentioned non-inferiority during registration, 78 did not mention non-inferiority during registration and we did not find evidence for registration for 13 trials. The median likelihood of degradation in these sub-groups was 0.51, 0.51 and 0.53 respectively.

### Proposal for a third margin, M3

Our concept of considering the likelihood of degradation allows to define a third margin M3, in addition to M1 and M2, aiming at such a protection. M3 is chosen just as that margin which limits the likelihood of degradation, i.e., the probability of having declared a harmful treatment as non-inferior in a NI trial is limited to lesser than 50% even if the pre study distribution is characterised by 69% negative effects. The narrowest of the three margins M1, M2 and M3 should then be used. Assuming a normal distribution of the true effect and for a given power, this NI margin M3 is a fixed constant in case of the continuous and survival outcomes while in case of binary outcomes, it depends on the prevalence. Table 4 shows the actual values of M3 and the necessary sample sizes [27]–[29] to obtain a power of 80% and 90% in case of a true effect of 0. It is to be noted that in the case of survival outcome, the value of M3 suggested is more liberal than what is typically used nowadays. M3 were determined by computing the likelihood of degradation on a grid of margin values with 100,000 simulations and interpolation of the results for each margin.

**Table 4:** Suggested NI margin M3 for different scales of trial outcomes.

Scale of the outcome variable (effect measure)	Prevalence in the control arm	80% power		90% power	
		M3	Requiredsample size**	M3	Requiredsample size**
Continuous (Cohen’s d)		–0.23	594	−0.2	1052
Survival (HR)		0.71	267*	0.75	507*
Binary (risk difference)	0.4	−0.09	622	−0.11	1242
	0.5	−0.1	544	−0.12	1048
	0.6	−0.1	568	−0.12	1006
	0.7	−0.09	596	−0.11	1086
	0.8	−0.07	694	−0.09	1370
	0.9	−0.04	1392	−0.05	2360

Sample sizes are calculated according to Farrington and Manning [28] (for binary outcome), Rothmann [29] (continuous) and Crisp and Curtis [27] (survival).

*overall number of events required.

**The sample size is calculated for a two-arm trial with 1∶1 randomisation comparing the lower bound of a two-sided 95% CI with the margin.

## Discussion

Degradation of treatment effects in NI trials have been a concern raised by many researchers [10], [11], [16]–[23], [30]. To protect against this degradation, the NI margin is ideally to be set in a stringent way by the PI of a trial. To decide whether a margin is stringent enough, we suggest requiring that the likelihood of degradation i.e., the probability of having declared a less efficient treatment as non-inferior in a successful NI trial, is lesser than 50% even if the pre-study distribution is characterised by 69% negative effects.

Using this principle, our investigation revealed frequent high likelihoods of degradation as a consequence of the regular usage of liberal NI margins among the current NI trials. Only two fifth of the current NI trials had a likelihood of degradation lower than 50% suggesting that the current choice of NI margins frequently implies an insufficient protection.

The first part of our study looking at the distribution of NI margins found the use of liberal margins in line with previous studies [10], [11], [31]. Lange and Freitag [10] in 2005 compiled the margins used in 332 published NI and equivalence trials conducted during 1990–2000 and it corresponded to an average Cohen’s d of −0.5 and an OR of 0.46. NI trials in our study representing the period beyond 2004, found a narrower margin of Cohen’s d of −0.42 and of an OR of 0.55. This may point to some improvement as compared to the previous decade. However, within the period covered by our investigation, no time trend could be found. A recent update on NI margins by Gayet-Ageron et al [11] found an average margin of Cohen’s d of −0.40 among 16 trials reporting continuous outcomes of the sampled 100 NI and equivalence trials done 2004–2009, very similar to our estimate. Soonawala et al [31] also reported an average NI margin of 0.76 when expressed as a relative risk among 33 binary outcome trials which is, however, not directly comparable to our results.

The practice of lenient choice of margins seem to be prevalent in many research areas, with a very few like cancer research and cardiology showing a better perspective in the choice of margins thus maintaining lower likelihoods of degradation. This could not be explained entirely by the general trend towards more stringent margins we observed, when a serious, life-threatening condition or a mortality outcome are considered. This general trend may be interpreted in a way that researchers tend to act in a responsible way by choosing stringent NI margins when it is most important. However, the wide variation of the margins, even in these areas, points to liberal choices made by some, even in risky areas. Medical fields such as psychiatry and ophthalmology, with most of the trials having a likelihood of degradation above 50%, are a concern as there may be insufficient protection against the risk of degradation.

With more than three decades of experience with non-inferiority or equivalence concepts, FDA [8] as well as EMEA [9] guides one towards the choice of margin by preservation of a fraction of the effect of the standard treatment over placebo (efficacy margin or M1) and clinical margin (minimum clinically acceptable difference or M2). Additional efforts are taken by the regulatory agencies in standardising the NI margins by expert committees in each specific medical field and implementation of well-justified margins through discussion with trial sponsors at the protocol phase [7], [8], [13], [32]. However, both the approaches do not aim at protection against degradation on an average.

Introducing M3 as an additional criterion for choosing the NI margin allows a PI to contribute to the control of risk of degradation on an average, assuming that the distribution of true treatment effects has no greater tendency to negativity than in our moderate scenario (of a normal distribution and 69% negativity). Two recent publications have actually pointed to a tendency in the opposite direction: Soonawala et al [31] studying the treatment effects among 170 published NI trials carried out 1991–2008 found that the average treatment effect among published NI trials to be around 0, and in a publication from our group [25], we estimated the average true treatment effect based on NI trials identified in trial registers and we too found a value close to 0. Even though there is some empirical evidence that the optimistic scenario may be today more realistic than the moderate one, it cannot be used as a general excuse for a PI not to aim at contributing to the control of risk of degradation on average. The two publications have taken a look over all medical fields; however, there may be specific medical fields which suffer from a negative average true treatment effect. There is also a possibility that the NI design may be used carelessly, with its growing popularity, resulting in a less favourable distribution. As a PI cannot just rely on all other PIs in the same medical field to be careful enough in choosing the NI design, there remains a need for each PI to contribute to the control by choosing an adequate margin in each NI trial. Our proposal for M3 may help to achieve this in an objective manner. However, it also depends on solving the subjective choice of the degree of negativity on average for which a control of the likelihood of degradation is desirable, by a consensus in the scientific community.

Our study is prone to some selection issues which include NI margins not traceable among some trials, published trials over-representing successful trials and the two sources of NI trials being somewhat arbitrary. Association between these and the choice of margin is unclear. Publication of studies in major journals or registration with the mention of NI design may indicate high quality associated with careful choice of margin. On the other hand, less careful choice may increase the chance of publication by increasing the chance of success of the trial. Another limitation was that we had to rely on the margins mentioned in the publication as there was no access to the original study protocols. We should also mention that our considerations implicitly assume that the constancy assumption [16] holds on average, i.e., that on average the efficacy of the comparator in the NI trials is similar to what has been observed in superiority trials establishing this comparator. Violations of this assumption could be a further source for degradation as pointed out by Everson- Stewart et al [30].

In conclusion, the concept of likelihood of degradation allows to judge the choice of margins with respect to their contribution to a protection against a degradation on an average independent of the outcome scale of the NI trial. Using this concept, we could demonstrate that the current use of margins among NI trials does not protect against the risk of degradation on an average. Though some feel that these restrictions are unnecessary and may prevent truly effective drugs with improved safety [33], we feel that the risk of degradation of treatment effects on an average should be taken seriously. Our considerations about a third hurdle M3 suggests that a protection is possible with sample sizes which are still feasible.

## Supporting Information

Figure S1 **Distribution of the likelihood of degradation among the current NI trials for the two variants of sensitivity analyses.** The diamonds represent the worst possible likelihood of degradation values and the dot represents the median likelihood of degradation in the moderate scenario.
(PDF)

Table S1 **Trial characteristics of Non-inferiority trials registered either in clinicaltrials.gov/ISRCTN (2000 to 2007) contributing to our analyses (N-62) and Non-inferiority trials published in the four major journals (2005 to 2011) contributing to our analyses (N-112).**
(PDF)

Table S2 **Data used for the estimation of likelihood of degradation in the non-inferiority trials registered either in clinicaltrials.gov/ISRCTN (2000 to 2007) contributing to our analyses (N-62) and non-inferiority trials published in the four major journals (2005 to 2011) contributing to our analyses (N-112).**
(PDF)

Table S3 **Scenarios of true effect distribution used in the calculation of likelihood of degradation in the sensitivity analyses.**
(PDF)
