# Supplementary material for: Choice of Non-Inferiority (NI) Margins Does Not Protect against Degradation of Treatment Effects on an Average – An Observational Study of Registered and Published NI Trials
Source: PLoS One. 2014 Jul 31;9(7):e103616. doi: 10.1371/journal.pone.0103616 (PMC4117500; doi:10.1371/journal.pone.0103616)

Main result:

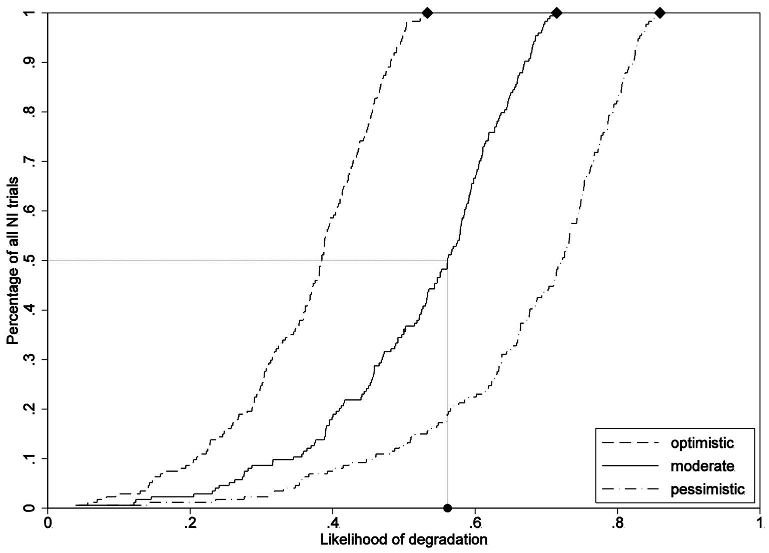

Sensitivity analysis - 1 (2/3rd of the original mean and sd):

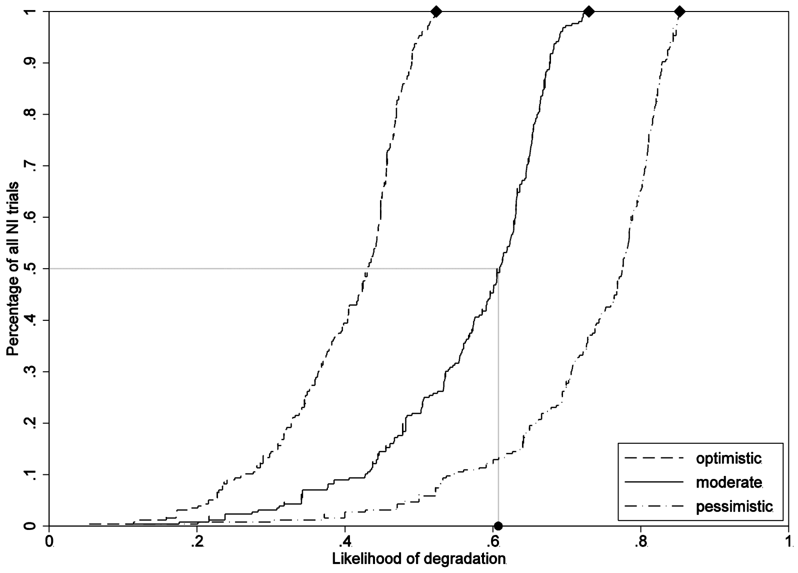

Sensitivity analysis - 2 (1.5 times the original mean and sd):

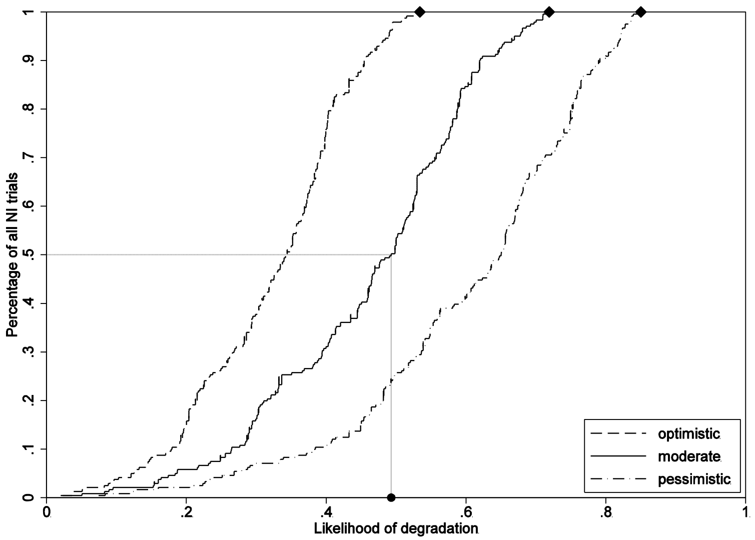

Supplement: Figure S1 — Distribution of the likelihood of degradation among the current NI trials for the two variants of sensitivity analyses. The diamonds represent the worst possible likelihood of degradation values and the dot represents the median likelihood of degradation in the moderate scenario. (PDF) [file pone.0103616.s001.pdf]
