# Supplementary material for: Choice of Non-Inferiority (NI) Margins Does Not Protect against Degradation of Treatment Effects on an Average – An Observational Study of Registered and Published NI Trials
Source: PLoS One. 2014 Jul 31;9(7):e103616. doi: 10.1371/journal.pone.0103616 (PMC4117500; doi:10.1371/journal.pone.0103616)
Supplement: Table S1 — Trial characteristics of Non-inferiority trials registered either in clinicaltrials.gov/ISRCTN (2000 to 2007) contributing to our analyses (N-62) and Non-inferiority trials published in the four major journals (2005 to 2011) contributing to our analyses (N-112). (PDF) [file pone.0103616.s002.pdf]

Supplementary table 1a: Trial characteristics of the Non-inferiority trials registered either in clinicaltrials.gov / ISRCTN (2000 to 2007) contributing to our analyses (N-62):

S.No.	Study id (reference)	Trial register id	Health/Disease condition studied in the trial	Type of drug/procedure/intervention	Comparison between	Primary outcome of non-inferiority in the trial
1	NI001(1)	NCT01163812	Gastric cancer	Gastrectomy, surgical procedure	Less invasive Laproscopic New vs Invasive Open Standard	Proportion of cases with no more than one missing lymph node station
2	NI004(2)	NCT00142350	Gastric cancer	Anti tumour chemotherapy agent	Oral New vs Intravenous Standard	One year overall survival
3	NI006(3)	NCT00527137	Anemia in chronic kidney disease	Erythropoiesis stimulating agent	New vs Standard	Change in serum Haemoglobin
4	NI008(4)	NCT00249223	Schizophrenia	Antipsychotic drug, route of administration	Injection New vs Oral Standard	Change in Positive and Negative syndrome scale (PANSS) score
5	NI011(5)	NCT00783744	Diabetes	Anti diabetic drug combination	New vs Standard	Change in HbA1c
6	NI023(6)	NCT00114608	Venous thromboembolism	Prophylactic procedure	New vs Standard	Change in blood flow velocity
7	NI024(7)	NCT00398684	HIV	Prophylactic drug	New vs Standard	Rate of transmission from mother to child
8	NI031(8)	NCT00051753	Otitis Media	Antibiotic drug	New vs Standard	Clinical cure rate
9	NI034(9)	NCT00950794	Asthma	Anti asthmatic drug	New vs Standard	Change in Peak Expiratory Flow

10	NI038(10)	NCT00751829	hypertension	Anti hypertensive drug	New vs Standard	Change in systolic blood pressure
11	NI039(11)	NCT00253955	Pneumonia	Antibiotic drug	New vs Standard	Clinical cure rate
12	NI0310(12)	NCT00383136	Acute myocardial infarction	Adjunctive therapy to angioplasty	New vs Standard	Rate of complete ST resolution
13	NI0311(13)	NCT00139815	Acute coronary syndrome	Anti coagulant drug	Safer New vs Standard	Rate of composite of hazard events
14	NI0313(14)	NCT00274612	hypertension	anti hypertensive drug	New vs Standard	Change in systolic and diastolic blood pressure
15	NI0315(15)	NCT00208299	Myocardial perfusion	Diagnostic procedure	Safer , Well tolerated New vs Standard	Agreement rate
16	NI0318(16)‡	NCT00058682	Candidemia	Antifungal drug	New vs Standard	Clinical success rate
17	NI0320(17)	NCT00106288	Candidemia	Antifungal drug	Safer New vs Standard	Treatment success rate
18	NI0323(18)	NCT00062803	Venous thromboembolism	Anti coagulant drug	Fixed dose new vs Monitored dose adjusted Standard	Incidence rate
19	NI0324(19)	NCT00070655	Stroke and Systemic thromboembolism	Anti coagulant drug	Fixed dose new vs Monitored dose adjusted Standard	Cumulative Incidence
20	NI0325(20)	NCT00334386	hypertension	Anti hypertensive drug	Prolonged release vs Immediate release	Change in diastolic blood pressure
21	NI115(21)	NCT00150735	Epilepsy	Treatment drug	Better tolerable New vs Standard	Seizure free rate
22	NI116(22)	NCT00093067	Bacteremia and endocarditis	Antibiotic drug	New vs Standard	Success rate

23	NI117(23)	NCT00439855	Coronary artery disease	Anticoagulant drug	Safer, Convenient New vs Standard	Incidence of composite ischaemic endpoint
24	NI1110(24)	NCT00118638	Chemotherapy induced anemia	Treatment drug	Less frequent New vs Frequent standard treatment regimen	Incidence of blood transfusion
25	NI1111(25)	NCT00206128	Schizophrenia	Treatment drug	Extended release vs Immediate release	Failure rate
26	NI1112(26)	NCT00254878	Attention Deficit Disorder	Treatment drug	Extended release vs Immediate release	Attention and deportment rating scale
27	NI1113(27)	NCT00311961	COPD exacerbation	Treatment drug	Oral vs Intravenous	Failure rate
28	NI1115(28)	NCT00093158	Acute coronary syndrome	Antithrombotic regimen	Safer New vs Standard	Incidence of composite ischaemic endpoint
29	NI1118(29)	NCT00240474	hypertension	antihypertensive drug	New vs Standard	Change in systolic blood pressure
30	NI1122(30)	NCT00682760	Blepharospasm	Treatment drug	New vs Standard	Proportion of improvement in spasm severity
31	NI1126(31)	NCT00484900	malaria	Anti malarial drug combination	Easy to use New vs Standard	Parasitological cure rate
32	NI127(32)	NCT00299403	Depression	Treatment procedure	Safer New vs Standard	Rate of partial remission
33	NI129(33)	NCT00358176	Postmenopausal osteoporosis	treatment drug	Less frequent New vs Frequent standard	Change in bone mineral density
34	NI1211(34)	NCT00279344	Cancer	Anti analgesic drug	Well controlled New vs Standard	Change in pain intensity rating scale
35	NI1219(35)	NCT00486694	malaria	Anti malarial drug	New vs Standard	Parasitological cure rate

36	NI1222(36)	NCT00117169	Pulmonary embolism	Diagnostic procedure	Single drug versus Combination	3 month thromboembolic risk
37	NI1223(37)	NCT00325637	Ischemic stroke	antihypertensive drug	New vs Standard	Change in cerebral blood flow
38	NI1230(38)	NCT00150046	cardiac transplantation	Immunosuppressant	New vs Standard	Creatinine clearance rate
39	NI1235(39)	NCT00208312	Myocardial perfusion	Diagnostic procedure	Safer Well tolerated New vs Standard	Agreement rate
40	NI1239(40)	NCT00158405	HIV	Treatment strategies	Non continuous New vs Continuous standard treatment strategy	Proportion of patients with CD4 count < 350 per mm3
41	NI1240(41)	NCT00088491	Schizophrenia	Treatment regimens	Long acting injection vs Oral	Exacerbation rate
42	NI1246(42)	NCT00094523	HIV	treatment regimens	New vs Standard	Proportion of HIV RNA < 400 copies/ml
43	NI1254(43)	NCT00298350	HIV	Treatment drug	New vs Standard	Change in HIV RNA level
44	NI1255(44)	NCT00375713	Dermatitis & Eczema	Anti allergic drug	New vs Standard	Pruritis severity score
45	NI1256(45)	NCT00650598	Knee arthroscopy procedure	Analgesic drug	New vs Standard	VAS pain scale
46	NI1259(46)	NCT01082471	Abdominal surgery	Anasthetic drug	Well tolerated New vs Standard	VRS-11 pain intensity score
47	NI1260(47)	NCT00299884	Dyslipidemia	Treatment drug	Well tolerated New vs Standard	Change in LDL cholesterol
48	NI1263(48)	NCT00399464	Benign prostatic hyperplasia	Treatment drug	New vs Standard	Change in I-PSS score
49	NI1265(49)	NCT00417872	Clostridium difficile Colitis	Antibiotic drug	Less expensive New vs Standard	Response rate

50	NI1270(50)	NCT00209300	Ulcerative colitis	treatment drug	Less frequent New vs Frequent standard	Remission rate
51	NI1278(51)	NCT00140049	Glaucoma	Anti glaucoma drug	Less frequent New vs Frequent standard	Change in intra ocular pressure
52	NI1301(52)	ISRCTN65487167	Osteoarthritis	Treatment procedure	More accessible treatment vs less accessible	Pain assessment visual score
53	NI1302(53)	ISRCTN51678883	Esophagogastric cancer	Treatment drug	Convenient, less toxic drug vs Standard drug	Overall survival
54	NI1303(54)	ISRCTN11611768	Chronic Obstructive Pulmonary Disease	Treatment procedure	More tolerable New procedure vs less tolerable Standard	Chronic respiratory questionnaire score
55	NI1305(55)	ISRCTN81306618	Malaria	Treatment drug	Cheaper New vs Standard drug	Cure rate
56	NI1312(56)	ISRCTN62825862	Acute MI	Treatment procedure	Safer New stent vs Standard stent	In-lesion late loss at 9 months
57	NI1315(57)	ISRCTN89458845	Aortailiac occlusive disease	Treatment procedure	New vs Standard	Restenosis rate
58	NI1317(58)	ISRCTN27914471	Malaria	Treatment drug	Cheap tolerable New vs Standard	Failure rate
59	NI1318(59)	ISRCTN87811512	Abortion	Treatment drug	Safer New vs Standard	Abortion rate
60	NI1319(60)	ISRCTN89831716	Fertilisation	Treatment procedure	Safer New vs Standard	Fertilisation rate
61	NI1320(61)	ISRCTN07576538	Malaria	Treatment drug	Easier to administer , fixed New vs Standard dosing procedure	Cure rate
62	NI1321(62)†	ISRCTN36773557	Parturition	Treatment drug	Cheaper, easy-to-store New vs Standard	Vaginal delivery in 24 hrs

‡ Trial registered by principal investigator as a non-inferiority trial but registered by sponsor with no reference to non-inferiority

† the two identical trials had a pooled analysis

Supplementary table 1b: Trial characteristics of the Non-inferiority trials published in the four major journals (2005 to 2011) contributing to our analyses (N-112):

**S.No.**	**Study id (reference)**	**Health/Disease condition studied in the trial**	**Type of drug/procedure/intervention**	**Comparison between**	**Primary outcome of non-inferiority in the trial**
1	MJ001(63)	Malaria	Anti malarial drug, form of tablet	Easy to use New vs Standard	PCR corrected 28-day cure rate
2	MJ002(64)	Diabetes	Anti diabetic drug combination	Less frequent New vs Standard	Change in HbAc
3	MJ003(65)	Diabetes	Anti diabetic drug	Less frequent New vs Standard	Change in HbAc
4	MJ004(66)	Stroke	Anti coagulant drug	Fixed dose new vs Monitored dose adjusted Standard	Incidence of stroke or embolism
5	MJ005(67)	Colorectal surgery	Surgical procedure	Better tolerable New vs Standard	Rate of leakage
6	MJ007(68)	Diabetes	Anti diabetic drug	Less frequent New vs Standard	Change in HbAc
7	MJ008(69)	Thromboprophylaxis - hip replacement	Anti coagulant drug	Oral New vs Standard	Rate of composite of thromboembolism and death
8	MJ009(70)	HIV	Treatment drug	New vs Standard	Proportion of RNA count <400/ml
9	MJ010(71)	Head Injury	Treatment procedure	Less time consuming New vs Standard	Rate of no recovery
10	MJ011(72)	Infertility	Treatment procedure	Less expensive, More tolerable New vs Standard	Proportion with Pregnancy at 1 yr
11	MJ012(73)	Atrial fibrillation	Treatment procedure	Better tolerable New vs Standard	Incidence of composite failure endpoint

12	MJ013(74)	Diabetes	Anti diabetic drug combination	Less adverse effect New vs Standard	Incidence of composite failure endpoint
13	MJ014(75)	Cancer	Treatment drug	Less toxic New vs Standard	Disease rate
14	MJ015(76)	HIV	Treatment drug	New vs Standard	Proportion with RNA count <400/ml
15	MJ016(77)	Psoriasis	Treatment procedure	Home based New vs Outpatient based Standard	Success rate of high Psoriasis severity score
16	MJ017(78)	Candidaemia	Treatment drug	New vs Standard	Proportion with Negative blood culture for candidaemia
17	MJ019(79)	HIV	Treatment drug combination	Better tolerable New vs Standard	Proportion with RNA count <50/ml
18	MJ020(80)	Obssessive compulsive disorder	Treatment procedure	More accessible New vs Standard	Change in score
19	MJ021(81)	Anemia	Treatment drug	Less frequent, less monitored New vs Standard	Change in Hb in g/L
20	MJ022(82)	HIV	Treatment drug combination	New vs Standard	Proportion with RNA count <400/ml
21	MJ023(83)	Epilepsy	Treatment drug	New vs Standard	Retention rate
22	MJ024(84)	Physiotherapy for Neck pain	Treatment procedure	Less time consuming New vs Standard	Change in score
23	MJ025(85)	antibiotic	Treatment procedure	Easy, better tolerable New vs Standard	Rate of renal scarring
24	MJ026(86)	HIV	Treatment drug combination	Less toxic New vs Standard	Proportion with RNA count <50/ml
25	MJ027(87)	Pneumonia	Treatment procedure	Easy, better tolerable New vs Standard	Clinical success rate
26	MJ028(88)	Meningitis	Treatment drug	More accessible New vs Standard	Failure rate
27	MJ029(89)	Cancer	Treatment procedure	Less adverse effect New vs Standard	Relapse free rate
28	MJ030(90)	Trypanosomiasis	Treatment drug combination	Less toxic, easy New vs Standard	Cure rate
29	MJ031(91)	Invasive Candidiasis	Treatment drug	Safer New vs Standard	Proportion with negative blood

					culture for candidaemia
30	MJ032(92)	Osteoporosis	Treatment drug	Less frequent New vs Standard	Percentage change in bone density
31	MJ034(93)	Stroke	Treatment drug	Less adverse effect New vs Standard	Recurrence rate
32	MJ035(94)	Cancer	Treatment drug combination	Lower cost, less toxic New vs Standard	Survival
33	MJ036(95)	Carotid artery stenosis	Treatment procedure	New vs Standard	Incidence of Stroke or death
34	MJ037(96)	Malaria	Anti malarial drug	More accessible, less costly New vs Standard	Failure rate
35	MJ038(97)	Coronary artery disease	Treatment procedure	Safer New vs Standard	Incidence of composite failure endpoint
36	MJ039(98)	Thromboprophylaxis - knee arthroplasty	Anti coagulant drug	Oral New vs Standard	Incidence of thrombosis or embolism or death
37	MJ042(99)	Depression	Treatment drug	Better tolerable New vs Standard	Change in depression score
38	MJ043(100)	Contraception	Contraceptive drug	Better tolerable New vs Standard	12 month severe flareup rate
39	MJ044(101)	Renal transplant	Treatment drug	Less adverse effect New vs Standard	6 month rejection rate
40	MJ045(102)	Cornonary heart disease	Treatment procedure	New vs Standard	Insegment late luminal loss
41	MJ046(103)	Colon cancer	Treatment drug	Less adverse effect New vs Standard	Disease free survival rate
42	MJ047(104)	Thromboprophylaxis – total knee replacement	Anti coagulant drug	Easy to use New vs Standard	Incidence of composite endpoint of thrombolism, embolism, death
43	MJ048(105)	Influenza	Prophylactic procedure	More accessible New vs Standard	Incidence rate
44	MJ049(106)	Pulmonary adeno carcinoma	Treatment drug	New vs Standard	12 month progression free survival rate
45	MJ050(107)	Chronic hepatitis	Treatment drug	New vs Standard	Therapeutic response rate
46	MJ053(108)	Coronary artery	Treatment drug	New vs Standard	Incidence of omposite of major adverse cardiac/cerebrovascular

		disease			events
47	MJ054(109)	Venous thrombo embolism (VTE)	Treatment drug	Less cost, easy to use New vs Standard	Recurrence rate of venous thromboembolism
48	MJ055(110)	Pulmonary embolism	Diagnostic procedure	More informative New vs Standard	Incidence of thromboembolism
49	MJ057(111)	Catheter related infection	Treatment procedure	Less cost, easy to use New vs Standard	Catheter colonisation rate
50	MJ058(112)	Lower respiratory tract infection	Treatment procedure	Easy, less adverse effect New vs Standard	Incidence of composite adverse outcomes
51	MJ060(113)	Coronary artery disease - stent	Treatment procedure	New vs Standard	In segment late loss
52	MJ061(114)	Heart failure	Treatment drug combination	Less adverse effect New vs Standard	Survival
53	MJ062(115)	Acute myocardial infarction	Treatment procedure	Less adverse effect, less costly New vs Standard	Recovery rate at 90 mts
54	MJ063(116)	Atrial fibrillation	Anti coagulant drug	Less adverse effect New vs Standard	Incidence of stroke or embolism
55	MJ064(117)	Deep vein thrombosis (DVT)	Treatment drug	Less monitored New vs Standard	Recurrence rate
56	MJ065(118)	Parkinsons	Treatment drug	New vs Standard	Rate of change of score
57	MJ066(119)	Hepatoblastoma	Treatment drug	Less toxic New vs Standard	Rate of resection
58	MJ067(120)	Acute VTE	Treatment drug	Less monitored New vs Standard	6 month recurrence rate
59	MJ068(121)	Intensive care unit (ICU)	Treatment procedure	Ease of use New vs Standard	Multiple organ dysfunction rate
60	MJ069(122)	Lupus nephritis	Treatment drug	Better tolerable, Oral New vs Standard	Rate of remission
61	MJ072(123)	Carotid stenosis	Treatment procedure	Less invasive New vs Std	Incidence of composite failure endpoint
62	MJ074(124)	Leishmaniasis	Treatment drug	Less expensive, less time consuming New vs Standard	Cure rate

63	MJ075(125)	Viral hepatitis	Treatment drug combination	Shorter duration New vs Standard	Virological response rate
64	MJ076(126)	DVT	Treatment drug	Less adverse effect New vs Standard	Recurrence rate
65	MJ078(127)	Prostate Cancer	Treatment procedure	Less adverse effect New vs Standard	Survival
66	MJ079(128)	breast cancer	Treatment drug combination	Easy to use New vs Standard	Recurrence rate
67	MJ081(129)	Percutaneous endoscopic gastrotomy	Treatment procedure	Simpler New vs Standard	Rate of wound infection
68	MJ082(130)	Diabetes	Treatment drug combination	Less adverse effect New vs Standard	Change in HbA1c
69	MJ083(131)	Breast cancer	Treatment procedure	Less adverse effect New vs Standard	Survival
70	MJ084(132)	Septic shock	Treatment procedure	Less cost, less time consuming New vs Standard	In-Hospital mortality rate
71	MJ085(133)	Hearing loss	Treatment procedure	Less adverse effect New vs Standard	Change in hearing threshhold
72	MJ086(134)	Atrial fibrillation	Anti coagulant drug	Less monitored, less adverse effect New vs Standard	Incidence of cardiovascular major event
73	MJ087(135)	Aortic valve replacement	Treatment procedure	Less invasive New vs Std	Survival
74	MJ088(136)	Hepatitis	Treatment drug	Shorter duration New vs Standard	Sustained virologic response rate
75	MJ089(137)	Atrial fibrillation	Anti coagulant drug	Less adverse effect New vs Standard	Incidence of stroke or systemic embolism
76	MJ090(138)	Coronary artery disease	Treatment procedure	New vs Standard	Cumulative event rate
77	MJ091(139)	Macular degeneration	Treatment drug	New vs Standard	Change in visual acuity
78	MJ092(140)	Atrial fibrillation	Anti coagulant drug	Less monitored, less adverse effect New vs Standard	Ischemic/hemorrhagic event rate
79	MJ094(141)	Ovarian cancer	Treatment procedure	New vs Standard	Survival
80	MJ095(142)	Breast cancer	Treatment procedure	Shorter duration New vs Standard	Risk of local breast cancer

					recurrence
81	MJ096(143)	Atrial fibrillation	Treatment procedure	Less adverse effect New vs Standard	Incidence of composite CV endpoint
82	MJ098(144)	VTE	Treatment drug	Less monitored New vs Standard	Recurrent rate of VTE
83	MJ099(145)	Latent TB infection	Treatment drug combination	Shorter duration New vs Standard	Cumulative rate of TB confirmed
84	MJ100(146)	Visceral Leishmaniasis	Treatment drug	Less frequent New vs Standard	Cure rate
85	MJ101(147)	Coronary artery disease	Treatment procedure	New vs Standard	Incidence of composite CV endpoint
86	MJ102(148)	ANCA vasculitis	Treatment drug	Safer New vs Standard	Rate of remission of vasculitis
87	MJ103(149)	thromboprophylaxis - hip replacement	Anti coagulant drug	Ease of use New vs Standard	Incidence of composite CV endpoint
88	MJ104(150)	Coronary artery stenosis	Treatment procedure	Safer New vs Standard	Incidence of composite CV endpoint
89	MJ105(151)	Cardiac transplantation	Treatment procedure	Tolerable, Safer New vs Standard	Cumulative rate of composite rejection endpoint
90	MJ106(152)	HIV	Treatment drug	Tolerable, Safer New vs Standard	Confirmed response rate
91	MJ107(153)	Prostate cancer	Treatment drug	Less toxic, easy New vs Standard	Time to skeletal related event
92	MJ108(154)	Appendicitis	Treatment procedure	Less invasive New vs Std	Incidence of post intervention peritonitis
93	MJ109(155)	Leishmaniasis	Treatment drug combination	Safer, shorter duration New vs Standard	Cure rate
94	MJ112(156)	HIV	Treatment drug	Tolerable, Safer New vs Standard	Confirmed response rate
95	MJ113(157)	H pylori eradication	Treatment drug combination	New vs Standard	Rate of H pylori eradication
96	MJ114(158)	Post partum Haemorrhage	Treatment drug	More accessible New vs Standard	Cessation of active bleeding at 20 min

97	MJ115(159)	Cerebral ischemia	Treatment drug	Safer New vs Standard	Incidence of composite CV endpoint
98	MJ116(160)	Acute pulmonary embolism	Treatment procedure	Less cost, less time consuming New vs Standard	Incidence of symptomatic recurrent VTE
99	MJ117(161)	Breast cancer	Treatment procedure	Safer, more tolerable New vs Std	5 yr local recurrence rate
100	MJ118(162)	Malaria	Treatment drug combination	Ease of use New vs Standard	ACPR response rate
101	MJ120(163)	HIV	Treatment procedure	Easily accessible New vs Standard	Cumulative treatment failure rate
102	MJ121(164)	Diabetes	Treatment procedure	Safer, convenient New vs Standard	Change in HbAc
103	MJ122(165)	Diabetes	Treatment drug	Better tolerable New vs Standard	Change in HbAc
104	MJ123(166)	Endometrial cancer	Treatment procedure	Less adversr effects New vs Standard	5 yr vaginal recurrence rate
105	MJ124(167)	HIV	Treatment procedure	Easy, more accessible New vs Standard	Incidence of composite HIV endpoint
106	MJ125(168)	Thromboprophylaxis - knee replacement	Anti coagulant drug	Easy, less adverse effect New vs Standard	Incidence of composite CV endpoint
107	MJ126(169)	Emergency contraception	Treatment drug	New vs Standard	Pregnancy rate
108	MJ127(170)	HIV	Treatment drug combination	Less adverse effect New vs Standard	Response rate
109	MJ128(171)	ICU	Treatment procedure	Shorter duration, less adverse effect New vs Standard	Mortality at day28
110	MJ129(172)	TB	Treatment procedure	Convenient, more compliant, less adverse effect New vs Standard	Cure rate
111	MJ131(173)	Post partum Haemorrhage	Treatment drug	More accessible New vs Standard	Rate of bleeding cessation
112	MJ132(174)	Syphilis	Treatment drug	Convenient New vs Standard	Cure rate

References:

1. Lee JH, Kim Y-W, Ryu KW, Lee JR, Kim CG, Choi IJ, et al. A phase-II clinical trial of laparoscopy-assisted distal gastrectomy with D2 lymph node dissection for gastric cancer patients. Annals of Surgical Oncology. 2007;14(11):3148–53.

2. Boku N, Yamamoto S, Fukuda H, Shirao K, Doi T, Sawaki A, et al. Fluorouracil versus combination of irinotecan plus cisplatin versus S-1 in metastatic gastric cancer: a randomised phase 3 study. Lancet Oncol. 2009;10:1063–9.

3. Warady BA, Arar MY, Lerner G, Nakanishi AM, Stehman-Breen C. Darbepoetin alfa for the treatment of anemia in pediatric patients with chronic kidney disease. Pediatr Nephrol. 2006;21:1144–52.

4. Chue P, Eerdekens M, Augustyns I, Lachaux B, Molcan P, Eriksson L, et al. Comparative efficacy and safety of long-acting risperidone and risperidone oral tablets. European Neuropsychopharmacology. 2005;15:111–7.

5. Janka HU, Kliebe-Frisch C, Plewe G, Schweitzer MA, Riddle MC, Yki-Jarvinen H. Comparison of basal insulin added to oral agents versus twice-daily premixed insulin as initial insulin therapy for type 2 diabetes. DIABETES CARE. 2005;28:254–9.

6. Czyrny JJ, Kaplan RE, Wilding GE, Purdy CH, Hirsh J. Electrical foot stimulation: a potential new method of deep venous thrombosis prophylaxis. Vascular. 18(1):20–7.

7. Lallemant M, Jourdain G, Le Coeur S, Mary JY, Ngo-Giang-Huong N, Koetsawang S, et al. Single-dose perinatal nevirapine plus standard zidovudine to prevent mother-to-child transmission of HIV-1 in Thailand. New Engl J Med. 2004;351:217–28.

8. Noel GJ, Blumer JL, Pichichero ME, Hedrick JA, Schwartz RH, Balis DA, et al. A randomized comparative study of levofloxacin versus amoxicillin/clavulanate for treatment of infants and young children with recurrent or persistent acute otitis media. Pediatr Infect Dis J. 2008;27(6):483–9.

9. GSK. CTR summary for MDC - 408DP-02 [Internet]. 2006. Available from: http://download.gsk-clinicalstudyregister.com/files/3019.pdf

10. Mallion J-M, Heagerty A, Laeis P. Systolic blood pressure reduction with olmesartan medoxomil versus nitrendipine in elderly patients with isolated systolic hypertension. Journal of Hypertension. 2007;25(10):2168–77.

11. Sanofi Aventis. Sanofi Aventis Company study document - HR355_3035 [Internet]. 2008. Available from: www.clinicalstudyresults.org/documents/company-study_4282_0.pdf

12. Marzocchi A, Manari A, Piovaccari G, Marrozzini C, Marra S, Magnavacchi P, et al. Randomized comparison between tirofiban and abciximab to promote complete ST-resolution in primary angioplasty: results of the facilitated angioplasty with tirofiban or abciximab (FATA) in ST-elevation myocardial infarction trial. European Heart Journal). 2008;29(24):2972–80.

13. Yusuf S, Mehta SR, Chrolavicius S, Afzal R, Pogue J, Granger CB, et al. Comparison of fondaparinux and enoxaparin in acute coronary syndromes. New Engl J Med. 2006;354(14):1464–76.

14. Williams B, Gosse P, Lowe L, Harper R. The prospective, randomized investigation of the safety and efficacy of telmisartan versus ramipril using ambulatory blood pressure monitoring (PRISMA I). Journal of Hypertension. 2006;24(1):193–200.

15. Astellas Pharma US I. Lexiscan - efficacy and image agreement [Internet]. 2008. Available from: http://www.lexiscan.com/about/extent.php

16. Reboli AC, Rotstein C, Pappas PG, Chapman SW, Kett DH, Kumar D, et al. Anidulafungin versus fluconazole for invasive candidiasis. New Engl J Med. 2007;356:2472–82.

17. Kuse E-R, Chetchotisakd P, Da Cunha CA, Ruhnke M, Barrios C, Raghunadharao D, et al. Micafungin versus liposomal amphotericin B for candidaemia and invasive candidosis: a phase III randomised double-blind trial. Lancet. 2007;369(9572):1519–27.

18. Bousser MG, Bouthier J, Buller HR, Cohen AT, Crijns H, Davidson BL, et al. Comparison of idraparinux with vitamin K antagonists for prevention of thromboembolism in patients with atrial fibrillation: a randomised, open-label, non-inferiority trial. Lancet. 2008;371:315–21.

19. Buller HR, Cohen AT, Davidson B, Decousus H, Gallus AS, Gent M, et al. Idraparinux versus standard therapy for venous thromboembolic disease. New Engl J Med. 2007;357(11):1094–104.

20. Roca-Cusachs A, Aracil-Vilar J, Calvo-Gómez C, Vaquer-Pérez J-V, Laporta-Crespo F, Rojas-Serrano M-J, et al. Clinical effects of torasemide prolonged release in mild-to-moderate hypertension: a randomized noninferiority trial versus torasemide immediate release. Cardiovascular Therapeutics. 2008;26(2):91–100.

21. Brodie MJ, Perucca E, Ryvlin P, Ben-Menachem E, Meencke H-J. Comparison of levetiracetam and controlled-release carbamazepine in newly diagnosed epilepsy. Neurology. 2007;68:402–8.

22. Fowler VG, Boucher HW, Corey GR, Abrutyn E, Karchmer AW, Rupp ME, et al. Daptomycin versus standard therapy for bacteremia and endocarditis caused by Staphylococcus aureus. New Engl J Med. 2006;355:653–65.

23. Bertel O, Ramsay D, Wettstein T, Kurz DJ, Stettler I, Straumann E, et al. Intravenous enoxaparin versus unfractionated heparin in unselected patients undergoing percutaneous coronary interventions: the Zurich enoxaparin versus unfractionated heparin in PCI study (ZEUS). EuroIntervention. 2010;6(3):407–12.

24. Canon JL, Vansteenkiste J, Bodoky G, Mateos MV, Bastit L, Ferreira J, et al. Randomized, double-blind, active-controlled trial of every-3-week darbepoetin alfa for the treatment of chemotherapy-induced anemia. Journal of the National Cancer Institute. 2006;98:273–84.

25. Moeller H-J, Johnson S, Mateva T, Brecher M, Svensson O, Miller F, et al. Evaluation of the feasibility of switching from immediate release quetiapine to extended release quetiapine fumarate in stable outpatients with schizophrenia. International Clinical Psychopharmocology. 2008;23:95–105.

26. Novartis. Clinical trial results database - CTRD_CRIT124DDE01_Jan1607 [Internet]. 2007. Available from: www.novartisclinicaltrials.com/webapp/.../displayFile.do?trialResult=2289

27. Jong YP, Uil SM, Grotjohan HP, Postma DS, Kerstjens HAM, Van den Berg JWK. Oral or IV prednisolone in the treatment of COPD exacerbations - A randomized, controlled, double-blind study. Chest. 2007;132:1741–7.

28. Stone GW, McLaurin BT, Cox DA, Bertrand ME, Lincoff AM, Moses JW, et al. Bivalirudin for patients with acute coronary syndromes. New Engl J Med. 2006;355:2203–16.

29. Boehringer Ingelheim International GmbH. Boehringer Ingelheim Trial Synopsis 502.400 [Internet]. 2004. Available from: http://trials.boehringer-ingelheim.com/res/trial/data/pdf/502.400_U04-2006_new.pdf

30. Yoon JS, Kim JC, Lee SY. Double-blind, randomized, comparative study of Meditoxin versus Botox in the treatment of essential blepharospasm. Korean J Ophthalmol. 2009;23(3):137–41.

31. Sagara I, Rulisa S, Mbacham W, Adam I, Sissoko K, Maiga H, et al. Efficacy and safety of a fixed dose artesunate-sulphamethoxypyrazine-pyrimethamine compared to artemether-lumefantrine for the treatment of uncomplicated falciparum malaria across Africa: a randomized multi-centre trial. Malaria J. 2009;8:-.

32. Hansen PEB, Ravnkilde B, Videbech P, Clemmensen K, Sturlason R, Reiner M, et al. Low-frequency repetitive transcranial magnetic stimulation inferior to electroconvulsive therapy in treating depression. J ECT. 2011;27(1):26–32.

33. Delmas PD, McClung MR, Zanchetta JR, Racewicz A, Roux C, Benhamou C-L, et al. Efficacy and safety of risedronate 150 mg once a month in the treatment of postmenopausal osteoporosis. Bone. 2008;42(1):36–42.

34. Kress HG, Laage D, Hoerauf KH, Nolte T, Heiskanen T, Peterson R, et al. A randomized, open, parallel group, multicenter trial to investigate analgesic efficacy and safety of a new transdermal fentanyl patch

compared to standard opioid treatment in cancer pain. Journal of Pain and Symptom Management. 2008;36:268–79.

35. Kolaczinski K, Durrani N, Rahim S, Rowland M. Sulfadoxine-pyrimethamine plus artesunate compared with chloroquine for the treatment of vivax malaria in areas co-endemic for Plasmodium falciparum and P. vivax: a randomised non-inferiority trial in eastern Afghanistan. T Roy Soc Trop Med H. 2007;101(11):1081–7.

36. Righini M, Le Gal G, Aujesky D, Roy P-M, Sanchez O, Verschuren F, et al. Diagnosis of pulmonary embolism by multidetector CT alone or combined with venous ultrasonography of the leg: a randomised non-inferiority trial. Lancet. 2008;371:1343–52.

37. Hong K-S, Kang D-W, Bae H-J, Kim YK, Han M-K, Park J-M, et al. Effect of cilnidipine vs losartan on cerebral blood flow in hypertensive patients with a history of ischemic stroke: a randomized controlled trial. Acta Beurologica Scandinavica. 2010;121:51–7.

38. Viganò M, Dengler T, Mattei MF, Poncelet A, Vanhaecke J, Vermes E, et al. Lower incidence of cytomegalovirus infection with everolimus versus mycophenolate mofetil in de novo cardiac transplant recipients: a randomized, multicenter study. Transplant Infect Dis. 2010;12(1):23–30.

39. Iskandrian AE, Bateman TM, Belardinelli L, Blackburn B, Cerqueira MD, Hendel RC, et al. Adenosine versus regadenoson comparative evaluation in myocardial perfusion imaging: results of the ADVANCE phase 3 multicenter international trial. Journal of Nuclear Cardiology. 14(5):645–58.

40. Danel C, Moh R, Chaix M-L, Gabillard D, Gnokoro J, Diby C-J, et al. Two-months-off, four-months-on antiretroviral regimen increases the risk of resistance, compared with continuous therapy: a randomized trial involving West African adults. J Indect Dis. 2009;199(1):66–76.

41. Kane JM, Detke HC, Naber D, Sethuraman G, Lin DY, Bergstrom RF, et al. Olanzapine Long-Acting Injection: A 24-Week, Randomized, Double-Blind Trial of Maintenance Treatment in Patients With Schizophrenia. American Journal of Psychiatry. 2010;167:181–9.

42. GSK. CTR Summary for MDC - ESS100290 [Internet]. 2007. Available from: http://download.gsk-clinicalstudyregister.com/files/19896.pdf

43. Zolopa AR, Berger DS, Lampiris H, Zhong L, Chuck SL, Enejosa JV, et al. Activity of Elvitegravir, a Once-Daily Integrase Inhibitor, against Resistant HIV Type 1: Results of a Phase 2, Randomized, Controlled, Dose-Ranging Clinical Trial. Journal of Infectious Diseases. 2010;201:814–22.

44. UCB I. Randomized Phase III Study to Evaluate the Efficacy and Safety of Xyzal® (Levocetirizine) vs Zyrtec® (Cetirizine) in Subjects With Dermatitis and Eczema - study results [Internet]. 2009. Available from: http://clinicaltrials.gov/ct2/show/results/NCT00375713

45. Pfizer INC. PHRMA web synopsis Valdecoxib A3471039 [Internet]. 2005. Available from: www.clinicalstudyresults.org/documents/company-study_746_0.pdf

46. Binning AR, Przesmycki K, Sowinski P, Morrison LMM, Smith TW, Marcus P, et al. A randomised controlled trial on the efficacy and side-effect profile (nausea/vomiting/sedation) of morphine-6-glucuronide versus morphine for post-operative pain relief after major abdominal surgery. Eur J Pain. 2011;15(4):402–8.

47. Kumar SS, Lahey KA, Day A, LaHaye SA. Comparison of the efficacy of administering a combination of ezetimibe plus fenofibrate versus atorvastatin monotherapy in the treatment of dyslipidemia. Lipids Health Dis. 2009;8:56.

48. Sanofi Aventis. Sanofi Aventis Company study document - EFC5791 [Internet]. 2008. Available from: http://www.clinicalstudyresults.org/documents/company-study_7515_0.pdf

49. Musher DM, Logan N, Hamill RJ, DuPont HL, Lentnek A, Gupta A, et al. Nitazoxanide for the treatment of Clostridium difficile colitis. Clinical Infectious Diseases. 2006;43:421–7.

50. Dignass AU, Bokemeyer B, Adamek H, Mross M, Vinter-Jensen L, Boerner N, et al. Mesalamine Once Daily Is More Effective Than Twice Daily in Patients With Quiescent Ulcerative Colitis. Clinical Gastroenterology and Hepatology. 2009;7:762–9.

51. Pfizer INC. PhRMA Clinical Study Synopsis Protocol A6641038 [Internet]. 2007. Available from: http://www.clinicalstudyresults.org/documents/company-study_2989_0.pdf

52. Hill J, Lewis M, Bird H. Do OA patients gain additional benefit from care from a clinical nurse specialist?--a randomized clinical trial. Rheumatology (Oxford, England). 2009 Jun;48:658–64.

53. Cunningham D, Starling N, Rao S, Iveson T, Nicolson M, Coxon F, et al. Capecitabine and oxaliplatin for advanced esophagogastric cancer. The New England journal of medicine. 2008 Jan 3;358:36–46.

54. Puhan MA, Busching G, Schunemann HJ, VanOort E, Zaugg C, Frey M. Interval versus continuous high-intensity exercise in chronic obstructive pulmonary disease: a randomized trial. Annals of internal medicine. 2006 Dec 5;145:816–25.

55. Valecha N, Phyo AP, Mayxay M, Newton PN, Krudsood S, Keomany S, et al. An open-label, randomised study of dihydroartemisinin-piperaquine versus artesunate-mefloquine for falciparum malaria in Asia. PloS one. 2010;5:e11880.

56. Van der Hoeven BL, Liem SS, Dijkstra J, Bergheanu SC, Putter H, Antoni ML, et al. Stent malapposition after sirolimus-eluting and bare-metal stent implantation in patients with ST-segment elevation myocardial infarction: acute and 9-month intravascular ultrasound results of the MISSION! intervention study. JACC. Cardiovascular interventions. 2008 Apr;1:192–201.

57. Mwipatayi BP, Thomas S, Wong J, Temple SEL, Vijayan V, Jackson M, et al. A comparison of covered vs bare expandable stents for the treatment of aortoiliac occlusive disease. Journal of Vascular Surgery. 2011;54:1561–1570.e1.

58. Smithuis F, Kyaw MK, Phe O, Aye KZ, Htet L, Barends M, et al. Efficacy and effectiveness of dihydroartemisinin-piperaquine versus artesunate-mefloquine in falciparum malaria: an open-label randomised comparison. Lancet. 2006 Jun 24;367:2075–85.

59. Von Hertzen H, Huong NT, Piaggio G, Bayalag M, Cabezas E, Fang AH, et al. Misoprostol dose and route after mifepristone for early medical abortion: a randomised controlled noninferiority trial. BJOG : an international journal of obstetrics and gynaecology. 2010 Sep;117:1186–96.

60. De Vos A, Van Landuyt L, Van Ranst H, Vandermonde A, D'Haese V, Sterckx J, et al. Randomized sibling-oocyte study using recombinant human hyaluronidase versus bovine-derived Sigma hyaluronidase in ICSI patients. Human reproduction (Oxford, England). 2008 Aug;23:1815–9.

61. Sirima SB, Tiono AB, Gansane A, Diarra A, Ouedraogo A, Konate AT, et al. The efficacy and safety of a new fixed-dose combination of amodiaquine and artesunate in young African children with acute uncomplicated Plasmodium falciparum. Malaria journal. 2009;8:48.

62. Calder AA, Loughney AD, Weir CJ, Barber JW. Induction of labour in nulliparous and multiparous women: a UK, multicentre, open-label study of intravaginal misoprostol in comparison with dinoprostone. BJOG : an international journal of obstetrics and gynaecology. 2008 Sep;115:1279–88.

63. Abdulla S, Sagara I, Borrmann S, D'Alessandro U, Gonzalez R, Hamel M, et al. Efficacy and safety of artemether-lumefantrine dispersible tablets compared with crushed commercial tablets in African infants and children with uncomplicated malaria: a randomised, single-blind, multicentre trial. Lancet. 2008;372(9652):1819–27.

64. Bretzel RG, Nuber U, Landgraf W, Owens DR, Bradley C, Linn T. Once-daily basal insulin glargine versus thrice-daily prandial insulin lispro in people with type 2 diabetes on oral hypoglycaemic agents (APOLLO): an open randomised controlled trial. Lancet. 2008;371(9618):1073–84.

65. Buse JB, Rosenstock J, Sesti G, Schmidt WE, Montanya E, Brett JH, et al. Liraglutide once a day versus exenatide twice a day for type 2 diabetes: a 26-week randomised, parallel-group, multinational, open-label trial (LEAD-6). Lancet. 2009;374(9683):39–47.

66. Connolly SJ, Ezekowitz MD, Yusuf S, Eikelboom J, Oldgren J, Parekh A, et al. Dabigatran versus Warfarin in Patients with Atrial Fibrillation. New Eng J Med. 2009;361(12):1139–51.

67. Contant CME, Hop WCJ, Van ‘t Sant HP, Oostvogel HJM, Smeets HJ, Stassen LPS, et al. Mechanical bowel preparation for elective colorectal surgery: a multicentre randomised trial. Lancet. 2007;370(9605):2112–7.

68. Drucker DJ, Buse JB, Taylor K, Kendall DM, Trautmann M, Zhuang D, et al. Exenatide once weekly versus twice daily for the treatment of type 2 diabetes: a randomised, open-label, non-inferiority study. Lancet. 2008;372(9645):1240–50.

69. Eriksson BI, Dahl OE, Rosencher N, Kurth AA, Van Dijk CN, Frostick SP, et al. Dabigatran etexilate versus enoxaparin for prevention of venous thromboembolism after total hip replacement: a randomised, double-blind, non-inferiority trial. Lancet. 2007;370(9591):949–56.

70. Gallant JE, DeJesus E, Arribas, Pozniak AL, Gazzard B, Campo RE, et al. Tenofovir DF, emtricitabine, and efavirenz vs. zidovudine, lamivudine, and efavirenz for HIV. New Eng J Med. 2006;354(3):251–60.

71. Af Geijerstam J-L, Oredsson S, Britton M, OCTOPUS Study Investigators. Medical outcome after immediate computed tomography or admission for observation in patients with mild head injury: randomised controlled trial. Brit Med J. 2006;333(7566):465–8.

72. Heijnen EMEW, Eijkemans MJC, Klerk C, Polinder S, Beckers NGM, Klinkert ER, et al. A mild treatment strategy for in-vitro fertilisation: a randomised non-inferiority trial. Lancet. 2007;369(9563):743–9.

73. Holmes DR, Reddy VY, Turi ZG, Doshi SK, Sievert H, Buchbinder M, et al. Percutaneous closure of the left atrial appendage versus warfarin therapy for prevention of stroke in patients with atrial fibrillation: a randomised non-inferiority trial. Lancet. 2009;374(9689):534–42.

74. Home PD, Pocock SJ, Beck-Nielsen H, Curtis PS, Gomis R, Hanefeld M, et al. Rosiglitazone evaluated for cardiovascular outcomes in oral agent combination therapy for type 2 diabetes (RECORD): a multicentre, randomised, open-label trial. Lancet. 2009;373(9681):2125–35.

75. Kim ES, Hirsh V, Mok T, Socinski MA, Gervais R, Wu Y-L, et al. Gefitinib versus docetaxel in previously treated non-small-cell lung cancer (INTEREST): a randomised phase III trial. Lancet. 2008;372(9652):1809–18.

76. Eron JJ, Yeni P, Gathe JJ, Estrada V, DeJesus E, Staszewski S, et al. The KLEAN study of fosamprenavir-ritonavir versus lopinavir-ritonavir, each in combination with abacavir-lamivudine, for initial treatment of HIV infection over 48 weeks: a randomised non-inferiority trial. Lancet. 2006;368(9534):476–82.

77. Koek MBG, Buskens E, Van Weelden H, Steegmans PHA, Bruijnzeel-Koomen CAFM, Sigurdsson V. Home versus outpatient ultraviolet B phototherapy for mild to severe psoriasis: pragmatic multicentre randomised controlled non-inferiority trial (PLUTO study). Brit Med J. 2009;338.

78. Kullberg BJ, Sobel JD, Ruhnke M, Pappas PG, Viscoli C, Rex JH, et al. Voriconazole versus a regimen of amphotericin B followed by fluconazole for candidaemia in non-neutropenic patients: a randomised non-inferiority trial. Lancet. 2005;366(9495):1435–42.

79. Lennox JL, DeJesus E, Lazzarin A, Pollard RB, Ramalho Madruga JV, Berger DS, et al. Safety and efficacy of raltegravir-based versus efavirenz-based combination therapy in treatment-naive patients with HIV-1 infection: a multicentre, double-blind randomised controlled trial. Lancet. 2009;374(9692):796–806.

80. Lovell K, Cox D, Haddock G, Jones C, Raines D, Garvey R, et al. Telephone administered cognitive behaviour therapy for treatment of obsessive compulsive disorder: randomised controlled non-inferiority trial. Brit Med J. 2006;333(7574):883–6.

81. Levin NW, Fishbane S, Valdes Canedo F, Zeig S, Nassar GM, Moran JE, et al. Intravenous methoxy polyethylene glycol-epoetin beta for haemoglobin control in patients with chronic kidney disease who are on dialysis: a randomised non-inferiority trial (MAXIMA). Lancet. 2007;370(9596):1415–21.

82. Madruga JV, Berger D, McMurchie M, Suter F, Banhegyi D, Ruxrungtham K, et al. Efficacy and safety of darunavir-ritonavir compared with that of lopinavir-ritonavir at 48 weeks in treatment-experienced, HIV-infected patients in TITAN: a randomised controlled phase III trial. Lancet. 2007;370(9581):49–58.

83. Marson AG, Al-Kharusi AM, Alwaidh M, Appleton R, Baker GA, Chadwick DW, et al. The SANAD study of effectiveness of carbamazepine, gabapentin, lamotrigine, oxcarbazepine, or topiramate for treatment of partial epilepsy: an unblinded randomised controlled trial. Lancet. 2007;369(9566):1000–15.

84. Moffett JAK, Jackson DA, Richmond S, Hahn S, Coulton S, Farrin A, et al. Randomised trial of a brief physiotherapy intervention compared with usual physiotherapy for neck pain patients: outcomes and patients' preference. Brit Med J. 2005;330(7482):75–78A.

85. Montini G, Toffolo A, Zucchetta P, Dall'Amico R, Gobber D, Calderan A, et al. Antibiotic treatment for pyelonephritis in children: multicentre randomised controlled non-inferiority trial. Brit Med J. 2007;335(7616):386–388A.

86. Molina J-M, Andrade-Villanueva J, Echevarria J, Chetchotisakd P, Corral J, David N, et al. Once-daily atazanavir/ritonavir versus twice-daily lopinavir/ritonavir, each in combination with tenofovir and erntricitabine, for management of antiretroviral-naive HIV-1-infected patients: 48 week efficacy and safety results of the CASTLE study. Lancet. 2008;372(9639):646–55.

87. El Moussaoui R, Borgie C, Van den Broek P, Hustinx WN, Bresser P, Van den Berk GEL, et al. Effectiveness of discontinuing antibiotic treatment after three days versus eight days in mild to moderate-severe community acquired pneumonia: randomised, double blind study. Brit Med J. 2006;332(7554):1355–8.

88. Nathan N, Borel T, Djibo A, Evans D, Djibo S, Corty JF, et al. Ceftriaxone as effective as long-acting chloramphenicol in short-course treatment of meningococcal meningitis during epidemics: a randomised non-inferiority study. Lancet. 2005;366(9482):308–13.

89. Oliver RTD, Mason MD, Mead GM, Maase H, Rustin GJS, Joffe JK, et al. Radiotherapy versus single-dose carboplatin in adjuvant treatment of stage I seminoma: a randomised trial. Lancet. 2005;366(9482):293–300.

90. Priotto G, Kasparion S, Mutombo W, Ngouama D, Ghorashian S, Arnold U, et al. Nifurtimox-eflornithine combination therapy for second-stage African Trypanosoma brucei gambiense trypanosomiasis: a multicentre, randomised, phase III, non-inferiority trial. Lancet. 2009;374(9683):56–64.

91. Reboli AC, Rotstein C, Pappas PG, Chapman SW, Kett DH, Kumar D, et al. Anidulafungin versus fluconazole for invasive candidiasis. New Eng J Med. 2007;356(24):2472–82.

92. Reid DM, Devogelaer J-P, Saag K, Roux C, Lau C-S, Reginster J-Y, et al. Zoledronic acid and risedronate in the prevention and treatment of glucocorticoid-induced osteoporosis (HORIZON): a multicentre, double-blind, double-dummy, randomised controlled trial. Lancet. 2009;373(9671):1253–63.

93. Sacco RL, Diener H-C, Yusuf S, Cotton D, Ounpuu S, Lawton WA, et al. Aspirin and extended-release dipyridamole versus clopidogrel for recurrent stroke. New Eng J Med. 2008;359(12):1238–51.

94. Seymour MT, Maughan TS, Ledermann JA, Topham C, James R, Gwyther SJ, et al. Different strategies of sequential and combination chemotherapy for patients with poor prognosis advanced colorectal cancer (MRC FOCUS): a randomised controlled trial. Lancet. 2007;370(9582):143–52.

95. Ringleb PA, Allenberg J, Berger J, Brueckmann H, Eckstein H-H, Fraedrich G, et al. 30 day results from the SPACE trial of stent-protected angioplasty versus carotid endarterectomy in symptomatic patients: a randomised non-inferiority trial. Lancet. 2006;368(9543):1239–47.

96. Zongo I, Dorsey G, Rouamba N, Tinto H, Dokomajilar C, T Guiguemde R, et al. Artemether-lumefantrine versus amodiaquine plus sulfadoxine-pyrimethamine for uncomplicated falciparum malaria in Burkina Faso: a randomised non-inferiority trial. Lancet. 2007;369(9560):491–8.

97. Windecker S, Serruys PW, Wandel S, Buszman P, Trznadel S, Linke A, et al. Biolimus-eluting stent with biodegradable polymer versus sirolimus-eluting stent with durable polymer for coronary revascularisation (LEADERS): a randomised non-inferiority trial. Lancet. 2008;372(9644):1163–73.

98. Turpie AGG, Lassen MR, Davidson BL, Bauer KA, Gent M, Kwong LM, et al. Rivaroxaban versus enoxaparin for thromboprophylaxis after total knee arthroplasty (RECORD4): a randomised trial. Lancet. 2009;373(9676):1673–80.

99. Szegedi A, Kohnen R, Dienel A, Kieser M. Acute treatment of moderate to severe depression with hypericum extract WS 5570 (St John's wort): randomised controlled double blind non-inferiority trial versus paroxetine. Brit Med J. 2005;330(7490):503–6.

100. Petri M, Kim MY, Kalunian KC, Grossman J, Hahn BH, Sammaritano LR, et al. Combined oral contraceptives in women with systemic lupus erythematosus. New Eng J Med. 2005;353(24):2550–8.

101. Vincenti F, Larsen C, Durrbach A, Wekerle T, Nashan B, Blancho G, et al. Costimulation blockade with belatacept in renal transplantation. New Eng J Med. 2005;353(8):770–81.

102. Dibra A, Kastrati A, Mehilli J, Pache J, Schuhlen H, Beckerath N, et al. Paclitaxel-eluting or sirolimus-eluting stents to prevent restenosis in diabetic patients. New Eng J Med. 2005;353(7):663–70.

103. Twelves C, Wong A, Nowacki MP, Abt M, Burris H, Carrato A, et al. Capecitabine as adjuvant treatment for stage III colon cancer. New Eng J Med. 2005;352(26):2696–704.

104. Lassen MR, Raskob GE, Gallus A, Pineo G, Chen D, Portman RJ, et al. Apixaban or Enoxaparin for Thromboprophylaxis after Knee Replacement. New Eng J Med. 2009;361(6):594–604.

105. Loeb M, Dafoe N, Mahony J, John M, Sarabia A, Glavin V, et al. Surgical Mask vs N95 Respirator for Preventing Influenza Among Health Care Workers A Randomized Trial. J Am Med Assoc. 2009;302(17):1865–71.

106. Mok TS, Wu Y-L, Thongprasert S, Yang C-H, Chu D-T, Saijo N, et al. Gefitinib or Carboplatin-Paclitaxel in Pulmonary Adenocarcinoma. New Eng J Med. 2009;361(10):947–57.

107. Lai C-L, Gane E, Liaw Y-F, Hsu C-W, Thongsawat S, Wang Y, et al. Telbivudine versus lamivudine in patients with chronic hepatitis B. New Eng J Med. 2007;357(25):2576–88.

108. Serruys PW, Morice M-C, Kappetein AP, Colombo A, Holmes DR, Mack MJ, et al. Percutaneous Coronary Intervention versus Coronary-Artery Bypass Grafting for Severe Coronary Artery Disease. New Eng J Med. 2009;360(10):961–72.

109. Kearon C, Ginsberg JS, Julian JA, Douketis J, Solymoss S, Ockelford P, et al. Comparison of fixed-dose weight-adjusted unfractionated heparin and low-molecular-weight heparin for acute treatment of venous thromboembolism. J Am Med Assoc. 2006;296(8):935–42.

110. Anderson DR, Kahn SR, Rodger MA, Kovacs MJ, Morris T, Hirsch A, et al. Computed tomographic pulmonary angiography vs ventilation-perfusion lung scanning in patients with suspected pulmonary embolism - A Randomized controlled trial. J Am Med Assoc. 2007;298(23):2743–53.

111. Timsit J-F, Schwebel C, Bouadma L, Geffroy A, Garrouste-Orgeas M, Pease S, et al. Chlorhexidine-Impregnated Sponges and Less Frequent Dressing Changes for Prevention of Catheter-Related Infections in Critically Ill Adults A Randomized Controlled Trial. J Am Med Assoc. 2009;301(12):1231–41.

112. Schuetz P, Christ-Crain M, Thomann R, Falconnier C, Wolbers M, Widmer I, et al. Effect of Procalcitonin-Based Guidelines vs Standard Guidelines on Antibiotic Use in Lower Respiratory Tract Infections The ProHOSP Randomized Controlled Trial. J Am Med Assoc. 2009;302(10):1059–66.

113. Stone GW, Midei M, Newman W, Sanz M, Hermiller JB, Williams J, et al. Comparison of an everolimus-eluting stent and a paclitaxel-eluting stent in patients with coronary artery disease - A randomized trial. J Am Med Assoc. 2008;299(16):1903–13.

114. Konstam MA, Gheorghiade M, Burnett JJC, Grinfeld L, Maggioni AP, Swedberg K, et al. Effects of oral tolvaptan in patients hospitalized for worsening heart failure - The EVEREST outcome trial. J Am Med Assoc. 2007;297(12):1319–31.

115. Valgimigli M, Campo G, Percoco G, Bolognese L, Vassanelli C, Colangelo S, et al. Comparison of angioplasty with infusion of tirofiban or abciximab and with implantation of sirolimus-eluting or uncoated stents for acute myocardial infarction - The MULTISTRATEGY randomized trial. J Am Med Assoc. 2008;299(15):1788–99.

116. Albers GW, Diener HC, Frison L, Grind M, Nevinson M, Partridge S, et al. Ximelagatran vs warfarin for stroke prevention in patients with nonvalvular atrial fibrillation - A Randomized trial. J Am Med Assoc. 2005;293(6):690–8.

117. Fiessinger JN, Huisman MV, Davidson BL, Bounameaux H, Francis CW, Eriksson H, et al. Ximelagatran vs low-molecular-weight heparin and warfarin for the treatment of deep vein thrombosis - A randomized trial. J Am Med Assoc. 2005;293(6):681–9.

118. Olanow CW, Rascol O, Hauser R, Feigin PD, Jankovic J, Lang A, et al. A Double-Blind, Delayed-Start Trial of Rasagiline in Parkinson’s Disease. New Eng J Med. 2009;361(13):1268–78.

119. Perilongo G, Maibach R, Shafford E, Brugieres L, Brock P, Morland B, et al. Cisplatin versus Cisplatin plus Doxorubicin for Standard-Risk Hepatoblastoma. New Eng J Med. 2009;361(17):1662–70.

120. Schulman S, Kearon C, Kakkar AK, Mismetti P, Schellong S, Eriksson H, et al. Dabigatran versus Warfarin in the Treatment of Acute Venous Thromboembolism. New Eng J Med. 2009;361(24):2342–52.

121. Lacroix J, Hebert PC, Hutchison JS, Hume HA, Tucci M, Ducruet T, et al. Transfusion strategies for patients in pediatric intensive care units. New Eng J Med. 2007;356(16):1609–19.

122. Ginzler EM, Dooley MA, Aranow C, Kim MY, Buyon J, Merrill JT, et al. Mycophenolate mofetil or intravenous cyclophosphamide for lupus nephritis. New Eng J Med. 2005;353(21):2219–28.

123. Mas J-L, Chatellier G, Beyssen B, Branchereau A, Moulin T, Becquemin J-P, et al. Endarterectomy versus stenting in patients with symptomatic severe carotid stenosis. New Eng J Med. 2006;355(16):1660–71.

124. Sundar S, Jha TK, Thakur CP, Sinha PK, Bhattacharya SK, Nguyen B, et al. Injectable paromomycin for visceral leishmaniasis in India. New Eng J Med. 2007;356(25):2571–81.

125. Shiffman ML, Suter F, Bacon BR, Nelson D, Harley H, Sola R, et al. Peginterferon alfa-2a and ribavirin for 16 or 24 weeks in HCV genotype 2 or 3. New Eng J Med. 2007;357(2):124–34.

126. Buller HR, Cohen AT, Davidson B, Decousus H, Gallus AS, Gent M, et al. Idraparinux versus standard therapy for venous thromboembolic disease. New Eng J Med. 2007;357(11):1094–104.

127. Bolla M, Reijke TM, Van Tienhoven G, Van den Bergh ACM, Oddens J, Poortmans PMP, et al. Duration of Androgen Suppression in the Treatment of Prostate Cancer. New Eng J Med. 2009;360(24):2516–27.

128. Muss HB, Berry DA, Cirrincione CT, Theodoulou M, Mauer AM, Kornblith AB, et al. Adjuvant Chemotherapy in Older Women with Early-Stage Breast Cancer. New Eng J Med. 2009;360(20):2055–65.

129. Blomberg J, Lagergren P, Martin L, Mattsson F, Lagergren J. Novel approach to antibiotic prophylaxis in percutaneous endoscopic gastrostomy (PEG): randomised controlled trial. Brit Med J. 2010;341.

130. Lund SS, Tarnow L, Frandsen M, Nielsen BB, Hansen BV, Pedersen O, et al. Combining insulin with metformin or an insulin secretagogue in non-obese patients with type 2 diabetes: 12 month, randomised, double blind trial. Brit Med J [Internet]. 2009 [cited 2013 Jan 10];339. Available from: http://www.ncbi.nlm.nih.gov/pmc/articles/PMC2775102/

131. Giuliano AE, Hunt KK, Ballman KV, Beitsch PD, Whitworth PW, Blumencranz PW, et al. Axillary Dissection vs No Axillary Dissection in Women With Invasive Breast Cancer and Sentinel Node Metastasis A Randomized Clinical Trial. J Am Med Assoc. 2011;305(6):569–75.

132. Jones AE, Shapiro NI, Trzeciak S, Arnold RC, Claremont HA, Kline JA, et al. Lactate Clearance vs Central Venous Oxygen Saturation as Goals of Early Sepsis Therapy A Randomized Clinical Trial. J Am Med Assoc. 2010;303(8):739–46.

133. Rauch SD, Halpin CF, Antonelli PJ, Babu S, Carey JP, Gantz BJ, et al. Oral vs Intratympanic Corticosteroid Therapy for Idiopathic Sudden Sensorineural Hearing Loss A Randomized Trial. J Am Med Assoc. 2011;305(20):2071–9.

134. Connolly S, Pogue J, Hart R, Pfeffer M, Hohnloser S, Chrolavicius S, et al. Clopidogrel plus aspirin versus oral anticoagulation for atrial fibrillation in the Atrial fibrillation Clopidogrel Trial with Irbesartan for prevention of Vascular Events (ACTIVE W): a randomised controlled trial. Lancet. 2006;367(9526):1903–12.

135. Smith CR, Leon MB, Mack MJ, Miller C, Moses JW, Svensson LG, et al. Transcatheter versus Surgical Aortic-Valve Replacement in High-Risk Patients. New Eng J Med. 2011;364(23):2187–98.

136. Sherman KE, Flamm SL, Afdhal NH, Nelson DR, Sulkowski MS, Everson GT, et al. Response-Guided Telaprevir Combination Treatment for Hepatitis C Virus Infection. New Eng J Med. 2011;365(11):1014–24.

137. Patel MR, Mahaffey KW, Garg J, Pan G, Singer DE, Hacke W, et al. Rivaroxaban versus Warfarin in Nonvalvular Atrial Fibrillation. New Eng J Med. 2011;365(10):883–91.

138. Park S-J, Kim Y-H, Park D-W, Yun S-C, Ahn J-M, Song HG, et al. Randomized Trial of Stents versus Bypass Surgery for Left Main Coronary Artery Disease. New Eng J Med. 2011;364(18):1718–27.

139. Martin DF, Maguire MG, Ying G, Grunwald JE, Fine SL, Jaffe GJ, et al. Ranibizumab and Bevacizumab for Neovascular Age-Related Macular Degeneration The CATT Research Group. New Eng J Med. 2011;364(20):1897–908.

140. Granger CB, Alexander JH, McMurray JJV, Lopes RD, Hylek EM, Hanna M, et al. Apixaban versus Warfarin in Patients with Atrial Fibrillation. New Eng J Med. 2011;365(11):981–92.

141. Vergote I, Trope CG, Amant F, Kristensen GB, Ehlen T, Johnson N, et al. Neoadjuvant Chemotherapy or Primary Surgery in Stage IIIC or IV Ovarian Cancer. New Eng J Med. 2010;363(10):943–53.

142. Whelan TJ, Pignol J-P, Levine MN, Julian JA, MacKenzie R, Parpia S, et al. Long-Term Results of Hypofractionated Radiation Therapy for Breast Cancer. New Eng J Med. 2010;362(6):513–20.

143. Van Gelder IC, Groenveld HF, Crijns HJ, Tuininga YS, Tijssen JG, Alings AM, et al. Lenient versus strict rate control in patients with atrial fibrillation. The New England journal of medicine. 2010 Apr 15;362:1363–73.

144. Bauersachs R, Berkowitz SD, Brenner B, Buller HR, Decousus H, Gallus AS, et al. Oral Rivaroxaban for Symptomatic Venous Thromboembolism. New Eng J Med. 2010;363(26):2499–510.

145. Sterling TR, Villarino ME, Borisov AS, Shang N, Gordin F, Bliven-Sizemore E, et al. Three Months of Rifapentine and Isoniazid for Latent Tuberculosis Infection. New Eng J Med. 2011;365(23):2155–66.

146. Sundar S, Chakravarty J, Agarwal D, Rai M, Murray HW. Single-Dose Liposomal Amphotericin B for Visceral Leishmaniasis in India. New Eng J Med. 2010;362(6):504–12.

147. Stone GW, Rizvi A, Newman W, Mastali K, Wang JC, Caputo R, et al. Everolimus-Eluting versus Paclitaxel-Eluting Stents in Coronary Artery Disease. New Eng J Med. 2010;362(18):1663–74.

148. Stone JH, Merkel PA, Spiera R, Seo P, Langford CA, Hoffman GS, et al. Rituximab versus Cyclophosphamide for ANCA-Associated Vasculitis. New Eng J Med. 2010;363(3):221–32.

149. Lassen MR, Gallus A, Raskob GE, Pineo G, Chen D, Ramirez LM, et al. Apixaban versus Enoxaparin for Thromboprophylaxis after Hip Replacement. New Eng J Med. 2010;363(26):2487–98.

150. Serruys PW, Silber S, Garg S, Van Geuns RJ, Richardt G, Buszman PE, et al. Comparison of Zotarolimus-Eluting and Everolimus-Eluting Coronary Stents. New Eng J Med. 2010;363(2):136–46.

151. Pham MX, Teuteberg JJ, Kfoury AG, Starling RC, Deng MC, Cappola TP, et al. Gene-Expression Profiling for Rejection Surveillance after Cardiac Transplantation. New Eng J Med. 2010;362(20):1890–900.

152. Cohen CJ, Andrade-Villanueva J, Clotet B, Fourie J, Johnson MA, Ruxrungtham K, et al. Rilpivirine versus efavirenz with two background nucleoside or nucleotide reverse transcriptase inhibitors in treatment-naive adults infected with HIV-1 (THRIVE): a phase 3, randomised, non-inferiority trial. Lancet. 2011;378(9787):229–37.

153. Fizazi K, Carducci M, Smith M, Damiao R, Brown J, Karsh L, et al. Denosumab versus zoledronic acid for treatment of bone metastases in men with castration-resistant prostate cancer: a randomised, double-blind study. Lancet. 2011;377(9768):813–22.

154. Vons C, Barry C, Maitre S, Pautrat K, Leconte M, Costaglioli B, et al. Amoxicillin plus clavulanic acid versus appendicectomy for treatment of acute uncomplicated appendicitis: an open-label, non-inferiority, randomised controlled trial. Lancet. 2011;377(9777):1573–9.

155. Sundar S, Sinha PK, Rai M, Verma DK, Nawin K, Alam S, et al. Comparison of short-course multidrug treatment with standard therapy for visceral leishmaniasis in India: an open-label, non-inferiority, randomised controlled trial. Lancet. 2011;377(9764):477–86.

156. Molina J-M, Cahn P, Grinsztejn B, Lazzarin A, Mills A, Saag M, et al. Rilpivirine versus efavirenz with tenofovir and emtricitabine in treatment-naive adults infected with HIV-1 (ECHO): a phase 3 randomised double-blind active-controlled trial. Lancet. 2011;378(9787):238–46.

157. Malfertheiner P, Bazzoli F, Delchier J-C, Celinski K, Giguere M, Riviere M, et al. Helicobacter pylori eradication with a capsule containing bismuth subcitrate potassium, metronidazole, and tetracycline given with omeprazole versus clarithromycin-based triple therapy: a randomised, open-label, non-inferiority, phase 3 trial. Lancet. 2011;377(9769):905–13.

158. Winikoff B, Dabash R, Durocher J, Darwish E, Ngoc NTN, Leon W, et al. Treatment of post-partum haemorrhage with sublingual misoprostol versus oxytocin in women not exposed to oxytocin during labour: a double-blind, randomised, non-inferiority trial. Lancet. 2010;375(9710):210–6.

159. Bousser M-G, Amarenco P, Chamorro A, Fisher M, Ford I, Fox KM, et al. Terutroban versus aspirin in patients with cerebral ischaemic events (PERFORM): a randomised, double-blind, parallel-group trial. Lancet. 2011;377(9782):2013–22.

160. Aujesky D, Roy P-M, Verschuren F, Righini M, Osterwalder J, Egloff M, et al. Outpatient versus inpatient treatment for patients with acute pulmonary embolism: an international, open-label, randomised, non-inferiority trial. Lancet. 2011;378(9785):41–8.

161. Vaidya JS, Joseph DJ, Tobias JS, Bulsara M, Wenz F, Saunders C, et al. Targeted intraoperative radiotherapy versus whole breast radiotherapy for breast cancer (TARGIT-A trial): an international, prospective, randomised, non-inferiority phase 3 trial. Lancet. 2010;376(9735):91–102.

162. Tshefu AK, Gaye O, Kayentao K, Thompson R, Bhatt KM, Sesay SSS, et al. Efficacy and safety of a fixed-dose oral combination of pyronaridine-artesunate compared with artemether-lumefantrine in children and adults with uncomplicated Plasmodium falciparum malaria: a randomised non-inferiority trial. Lancet. 2010;375(9724):1457–67.

163. Sanne I, Orrell C, Fox MP, Conradie F, Ive P, Zeinecker J, et al. Nurse versus doctor management of HIV-infected patients receiving antiretroviral therapy (CIPRA-SA): a randomised non-inferiority trial. Lancet. 2010;376(9734):33–40.

164. Rosenstock J, Lorber DL, Gnudi L, Howard CP, Bilheimer DW, Chang P-C, et al. Prandial inhaled insulin plus basal insulin glargine versus twice daily biaspart insulin for type 2 diabetes: a multicentre randomised trial. Lancet. 2010;375(9733):2244–53.

165. Pratley RE, Nauck M, Bailey T, Montanya E, Cuddihy R, Filetti S, et al. Liraglutide versus sitagliptin for patients with type 2 diabetes who did not have adequate glycaemic control with metformin: a 26-week, randomised, parallel-group, open-label trial. Lancet. 2010;375(9724):1447–56.

166. Nout RA, Smit VTHBM, Putter H, Juergenliemk-Schulz IM, Jobsen JJ, Lutgens LCHW, et al. Vaginal brachytherapy versus pelvic external beam radiotherapy for patients with endometrial cancer of high-intermediate risk (PORTEC-2): an open-label, non-inferiority, randomised trial. Lancet. 2010;375(9717):816–23.

167. Mugyenyi P, Walker AS, Hakim J, Munderi P, Gibb DM, Kityo C, et al. Routine versus clinically driven laboratory monitoring of HIV antiretroviral therapy in Africa (DART): a randomised non-inferiority trial. Lancet. 2010;375(9709):123–31.

168. Lassen MR, Raskob GE, Gallus A, Pineo G, Chen D, Hornick P, et al. Apixaban versus enoxaparin for thromboprophylaxis after knee replacement (ADVANCE-2): a randomised double-blind trial. Lancet. 2010;375(9717):807–15.

169. Glasier AF, Cameron ST, Fine PM, Logan SJS, Casale W, Van Horn J, et al. Ulipristal acetate versus levonorgestrel for emergency contraception: a randomised non-inferiority trial and meta-analysis. Lancet. 2010;375(9714):555–62.

170. Eron JJ, Young B, Cooper DA, Youle M, DeJesus E, Andrade-Villanueva J, et al. Switch to a raltegravir-based regimen versus continuation of a lopinavir-ritonavir-based regimen in stable HIV-infected patients with suppressed viraemia (SWITCHMRK 1 and 2): two multicentre, double-blind, randomised controlled trials. Lancet. 2010;375(9712):396–407.

171. Bouadma L, Luyt C-E, Tubach F, Cracco C, Alvarez A, Schwebel C, et al. Use of procalcitonin to reduce patients' exposure to antibiotics in intensive care units (PRORATA trial): a multicentre randomised controlled trial. Lancet. 2010;375(9713):463–74.

172. Lienhardt C, Cook SV, Burgos M, Yorke-Edwards V, Rigouts L, Anyo G, et al. Efficacy and Safety of a 4-Drug Fixed-Dose Combination Regimen Compared With Separate Drugs for Treatment of Pulmonary Tuberculosis The Study C Randomized Controlled Trial. J Am Med Assoc. 2011;305(14):1415–23.

173. Blum J, Winikoff B, Raghavan S, Dabash R, Ramadan MC, Dilbaz B, et al. Treatment of post-partum haemorrhage with sublingual misoprostol versus oxytocin in women receiving prophylactic oxytocin: a double-blind, randomised, non-inferiority trial. Lancet. 2010;375(9710):217–23.

174. Riedner G, Rusizoka M, Todd J, Maboko L, Hoelscher M, Mmbando D, et al. Single-Dose Azithromycin versus Penicillin G Benzathine for the Treatment of Early Syphilis. New Eng J Med. 2005;353(12):1236–44.
