# Supplementary material for: Choice of Non-Inferiority (NI) Margins Does Not Protect against Degradation of Treatment Effects on an Average – An Observational Study of Registered and Published NI Trials
Source: PLoS One. 2014 Jul 31;9(7):e103616. doi: 10.1371/journal.pone.0103616 (PMC4117500; doi:10.1371/journal.pone.0103616)
Supplement: Table S2 — Data used for the estimation of likelihood of degradation in the non-inferiority trials registered either in clinicaltrials.gov/ISRCTN (2000 to 2007) contributing to our analyses (N-62) and non-inferiority trials published in the four major journals (2005 to 2011) contributing to our analyses (N-112). (PDF) [file pone.0103616.s003.pdf]

Supplementary table 2a: Data used for the estimation of likelihood of harm in the Non-inferiority trials registered either in clinicaltrials.gov / ISRCTN (2000 to 2007) contributing to our analyses (N-62):

S.No.	Study id	Overall Sample size	Scale of the primary outcome variable	Non-inferiority Margin*	Response rate in the control arm‡	Number of events in the control arm†
1	NI001	164	Binary	-0.1	0.55	
2	NI004	466	Time to event	0.86		204
3	NI006	86	Continuous	-0.61		
4	NI008	541	Continuous	-0.56		
5	NI011	364	Continuous	-0.47		
6	NI023	80	Continuous	-0.51		
7	NI024	1264	Binary	-0.025	0.96	
8	NI031	1305	Binary	-0.1	0.80	
9	NI034	341	Continuous	-0.54		
10	NI038	382	Continuous	-0.35		
11	NI039	256	Binary	-0.15	0.81	
12	NI0310	681	Binary	-0.1	0.50	
13	NI0311	20078	Time to event	0.84		573
14	NI0313	801	Continuous	-0.25		
15	NI0315	1113	Binary	-0.13	0.61	
16	NI0318	245	Binary	-0.2	0.70	
17	NI0320	392	Binary	-0.15	0.65	
18	NI0323	2215	Binary	-0.015	0.96	
19	NI0324	4576	Time to event	0.67		27
20	NI0325	380	Continuous	-0.31		
21	NI115	472	Binary	-0.15	0.45	
22	NI116	235	Binary	-0.2	0.65	
23	NI117	876	Time to event	0.33		31
24	NI1110	672	Binary	-0.125	0.70	

25	NI1111	393	Binary	-0.06	0.94	
26	NI1112	294	Continuous	-0.58		
27	NI1113	210	Binary	-0.15	0.63	
28	NI1115	9215	Binary	-0.018	0.94	
29	NI1118	872	Continuous	-0.25		
30	NI1122	60	Binary	-0.15	0.86	
31	NI1126	827	Binary	-0.06	0.94	
32	NI127	60	Binary	-0.3	0.57	
33	NI129	1051	Continuous	-0.46		
34	NI1211	173	Continuous	-0.54		
35	NI1219	180	Binary	-0.14	0.96	
36	NI1222	1276	Binary	-0.015	0.99	
37	NI1223	167	Continuous	-0.25		
38	NI1230	176	Continuous	-0.26		
39	NI1235	758	Binary	-0.13	0.64	
40	NI1239	422	Binary	-0.15	0.70	
41	NI1240	603	Binary	-0.2	0.93	
42	NI1246	304	Binary	-0.12	0.93	
43	NI1254	134	Continuous	-0.49		
44	NI1255	340	Binary	-0.1	0.78	
45	NI1256	283	Continuous	-0.54		
46	NI1259	517	Continuous	-0.24		
47	NI1260	43	Binary	-0.2	0.37	
48	NI1263	938	Continuous	-0.18		
49	NI1265	110	Binary	-0.15	0.82	
50	NI1270	353	Binary	-0.1	0.60	
51	NI1278	238	Continuous	-0.67		
52	NI1301	79	Continuous	-0.50		
53	NI1302	964	Time to event	0.81		438

54	NI1303	86	Continuous	-0.58		
55	NI1305	1148	Binary	-0.005	0.92	
56	NI1312	255	Continuous	-0.70		
57	NI1315	159	Time to event	0.91		30
58	NI1317	652	Binary	-0.1	0.95	
59	NI1318	2962	Binary	-0.03	0.95	
60	NI1319	1232	Binary	-0.1	0.93	
61	NI1320	682	Binary	-0.05	0.95	
62	NI1321	619	Binary	-0.1	0.47	

*non-inferiority margin expressed based on the scale of the primary outcome in the trial - as risk difference for binary, Cohen’s d for continuous and hazard ratio for time to event outcomes
‡ used in trials where the primary outcome is binary
† used in trials where the primary outcome is time to event

Supplementary table 2b: Data used for the estimation of likelihood of harm in the Non-inferiority trials published in the four major journals (2005 to 2011) contributing to our analyses (N-112):

S.No.	Study id	Overall Sample size	Scale of the primary outcome variable	Non-inferiority Margin*	Treatment effect in the control arm‡	Number of events in the control arm†
1	MJ001	812	Binary	-0.05	0.95	
2	MJ002	412	Continuous	-0.40		
3	MJ003	464	Continuous	-0.33		
4	MJ004	18113	Binary	-0.0077	0.98	
5	MJ005	1354	Binary	-0.03	0.95	
6	MJ007	295	Continuous	-0.33		
7	MJ008	2651	Binary	-0.077	0.80	
8	MJ009	487	Binary	-0.13	0.73	
9	MJ010	2525	Binary	-0.05	0.75	
10	MJ011	404	Binary	-0.125	0.45	
11	MJ012	707	Time to event	0.50		18
12	MJ013	4447	Time to event	0.83		323
13	MJ014	1433	Time to event	0.87		576
14	MJ015	838	Binary	-0.12	0.70	
15	MJ016	185	Binary	-0.15	0.85	
16	MJ017	370	Binary	-0.15	0.65	
17	MJ019	521	Binary	-0.12	0.75	
18	MJ020	72	Continuous	-0.58		
19	MJ021	540	Continuous	-0.61		
20	MJ022	419	Binary	-0.12	0.68	
21	MJ023	1609	Binary	-0.1	0.70	
22	MJ024	221	Continuous	-0.12		
23	MJ025	400	Binary	-0.1	0.85	
24	MJ026	883	Binary	-0.1	0.70	

25	MJ027	114	Binary	-0.1	0.93	
26	MJ028	308	Binary	-0.1	0.85	
27	MJ029	1477	Binary	-0.03	0.96	
28	MJ030	286	Binary	-0.1	0.93	
29	MJ031	245	Binary	-0.2	0.70	
30	MJ032	771	Continuous	-0.12		
31	MJ034	20332	Time to event	0.93		898
32	MJ035	2135	Time to event	0.85		606
33	MJ036	1183	Binary	-0.025	0.95	
34	MJ037	748	Binary	-0.03	0.96	
35	MJ038	1707	Binary	-0.04	0.92	
36	MJ039	1742	Binary	-0.04	0.73	
37	MJ042	244	Continuous	-0.29		
38	MJ043	183	Binary	-0.09	0.94	
39	MJ044	140	Binary	-0.2	0.85	
40	MJ045	205	Continuous	-0.36		
41	MJ046	1987	Time to event	0.80		380
42	MJ047	3195	Binary	-0.056	0.84	
43	MJ048	422	Binary	-0.09	0.80	
44	MJ049	1217	Time to event	0.83		497
45	MJ050	1373	Binary	-0.15	0.67	
46	MJ053	1740	Binary	-0.066	0.88	
47	MJ054	697	Binary	-0.05	0.94	
48	MJ055	1406	Binary	-0.025	0.84	
49	MJ057	1636	Binary	-0.03	0.88	
50	MJ058	1359	Binary	-0.075	0.80	
51	MJ060	1001	Continuous	-0.42		
52	MJ061	1080	Binary	-0.065	0.74	
53	MJ062	745	Binary	-0.105	0.85	

54	MJ063	6405	Binary	-0.02	0.96	
55	MJ064	2489	Binary	-0.04	0.96	
56	MJ065	910	Continuous	-0.35		
57	MJ066	235	Binary	-0.1	0.93	
58	MJ067	2539	Binary	-0.036	0.98	
59	MJ068	637	Binary	-0.1	0.88	
60	MJ069	140	Binary	-0.1	0.60	
61	MJ072	520	Binary	-0.02	0.94	
62	MJ074	666	Binary	-0.1	0.99	
63	MJ075	1319	Binary	-0.063	0.80	
64	MJ076	2215	Binary	-0.037	0.96	
65	MJ078	230	Time to event	0.74		98
66	MJ079	633	Time to event	0.80		291
67	MJ081	234	Binary	-0.15	0.80	
68	MJ082	101	Continuous	-0.63		
69	MJ083	856	Time to event	0.77		52
70	MJ084	300	Binary	-0.1	0.75	
71	MJ085	250	Continuous	-0.46		
72	MJ086	3335	Time to event	0.84		165
73	MJ087	699	Binary	-0.075	0.68	
74	MJ088	321	Binary	-0.105	0.90	
75	MJ089	14264	Time to event	0.68		241
76	MJ090	600	Binary	-0.07	0.87	
77	MJ091	549	Continuous	-0.38		
78	MJ092	18201	Binary	-0.006	0.98	
79	MJ094	670	Time to event	0.80		230
80	MJ095	1234	Binary	-0.05	0.93	
81	MJ096	614	Binary	-0.1	0.75	
82	MJ098	3449	Time to event	0.50		51

83	MJ099	5858	Binary	-0.0075	0.99	
84	MJ100	412	Binary	-0.0008	0.99	
85	MJ101	3687	Binary	-0.031	0.92	
86	MJ102	197	Binary	-0.2	0.30	
87	MJ103	3866	Binary	-0.014	0.94	
88	MJ104	2245	Binary	-0.035	0.92	
89	MJ105	602	Binary	-0.021	0.95	
90	MJ106	678	Binary	-0.12	0.75	
91	MJ107	950	Time to event	0.90		384
92	MJ108	239	Binary	-0.1	0.98	
93	MJ109	316	Binary	-0.07	0.97	
94	MJ112	690	Binary	-0.12	0.75	
95	MJ113	339	Binary	-0.1	0.80	
96	MJ114	978	Binary	-0.06	0.88	
97	MJ115	19100	Time to event	0.95		1062
98	MJ116	339	Binary	-0.04	0.99	
99	MJ117	2232	Binary	-0.025	0.94	
100	MJ118	1170	Binary	-0.05	0.93	
101	MJ120	812	Time to event	0.71		179
102	MJ121	448	Continuous	-0.38		
103	MJ122	439	Continuous	-0.42		
104	MJ123	427	Binary	-0.06	0.98	
105	MJ124	3316	Time to event	0.85		356
106	MJ125	3009	Binary	-0.04	0.84	
107	MJ126	2221	Binary	-0.0065	0.99	
108	MJ127	348	Binary	-0.12	0.88	
109	MJ128	621	Binary	-0.1	0.65	
110	MJ129	1348	Binary	-0.04	0.91	
111	MJ131	809	Binary	-0.06	0.88	

112	MJ132	155	Binary	-0.075	0.95

*non-inferiority margin expressed based on the scale of the primary outcome in the trial - as risk difference for binary, Cohen’s d for continuous and hazard ratio for time to event outcomes
‡ used in trials where the primary outcome is binary
† used in trials where the primary outcome is time to event
