# Supplementary material for: Choice of Non-Inferiority (NI) Margins Does Not Protect against Degradation of Treatment Effects on an Average – An Observational Study of Registered and Published NI Trials
Source: PLoS One. 2014 Jul 31;9(7):e103616. doi: 10.1371/journal.pone.0103616 (PMC4117500; doi:10.1371/journal.pone.0103616)
Supplement: Table S3 — Scenarios of true effect distribution used in the calculation of likelihood of degradation in the sensitivity analyses. (PDF) [file pone.0103616.s004.pdf]

Table S3: Scenarios of true effect distribution used in the calculation of likelihood of degradation in the sensitivity analyses:

**True effect distribution**	**Optimistic scenario**			**Moderate scenario**			**Pessimistic scenario**		
**% true treatment effect being positive**	**0.5**			**0.31**			**0.16**		
**Sensitivity analysis set**	***0.667**	**Actual**	***1.5**	***0.667**	**Actual**	***1.5**	***0.667**	**Actual**	***1.5**
Continuous outcome (Cohen‘s d)									
Average true treatment effect	0	0	0	-0.0335	-0.05	-0.075	-0.067	-0.1	-0.15
Standard deviation	0.067	0.1	0.15	0.067	0.1	0.15	0.067	0.1	0.15
Binary outcome (log odds ratio)									
Average true treatment effect	0	0	0	-0.06801	-0.1015	-0.1523	-0.136	-0.203	-0.305
Standard deviation	0.136	0.203	0.305	0.13601	0.203	0.3045	0.13601	0.203	0.3045
Average true treatment effect as OR	1	1	1	0.93	0.90	0.86	0.87	0.82	0.74
Survival outcome (log hazards ratio)									
Average true treatment effect	0	0	0	-0.04824	-0.072	-0.108	-0.0965	-0.144	-0.216
Standard deviation	0.0965	0.144	0.216	0.09648	0.144	0.216	0.09648	0.144	0.216
Average true treatment effect as HR	1	1	1	0.95	0.93	0.90	0.91	0.87	0.81
Median likelihood of degradation	0.43	0.39	0.34	0.62	0.56	0.49	0.78	0.72	0.65
